# Supplementary material for: City size and the spreading of COVID-19 in Brazil
Source: PLoS One. 2020 Sep 23;15(9):e0239699. doi: 10.1371/journal.pone.0239699 (PMC7510961; doi:10.1371/journal.pone.0239699)
Supplement: S1 Appendix — (PDF) [file pone.0239699.s001.pdf]

S1 Appendix for

City size and the spreading of COVID-19 in Brazil

Haroldo V. Ribeiro, Andre S. Sunahara, Jack Sutton, Matjaž Perc, and Quentin S. Hanley

PLOS ONE, 2020

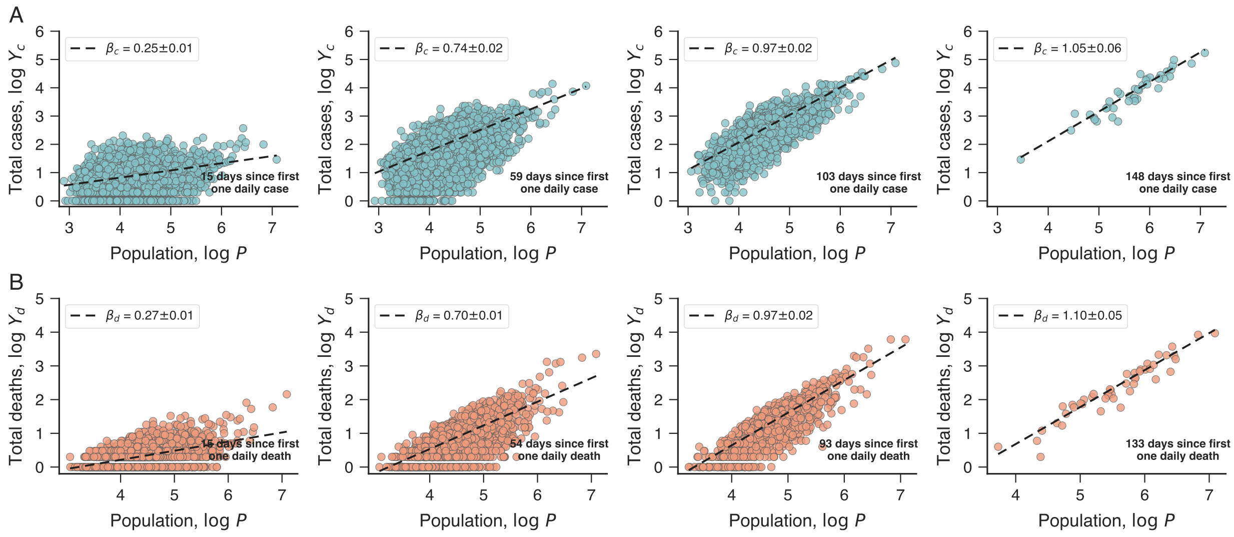

Figure 1. **Urban scaling relations of COVID-19 cases and deaths under different choices of values for the number of daily cases or daily deaths as reference points.** The same plots of Figure 1 in the main text but considering the first one daily case and first one daily death as reference points.

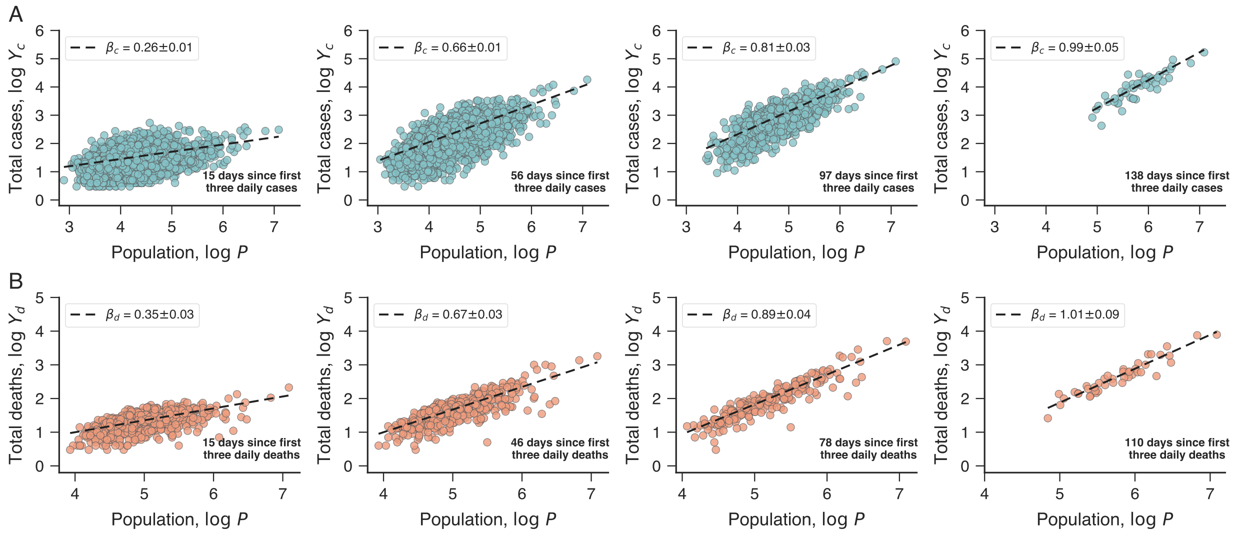

Figure 2. **Urban scaling relations of COVID-19 cases and deaths under different choices of values for the number of daily cases or daily deaths as reference points.** The same plots of Figure 1 in the main text but considering the first three daily cases and the first three daily deaths as reference points.

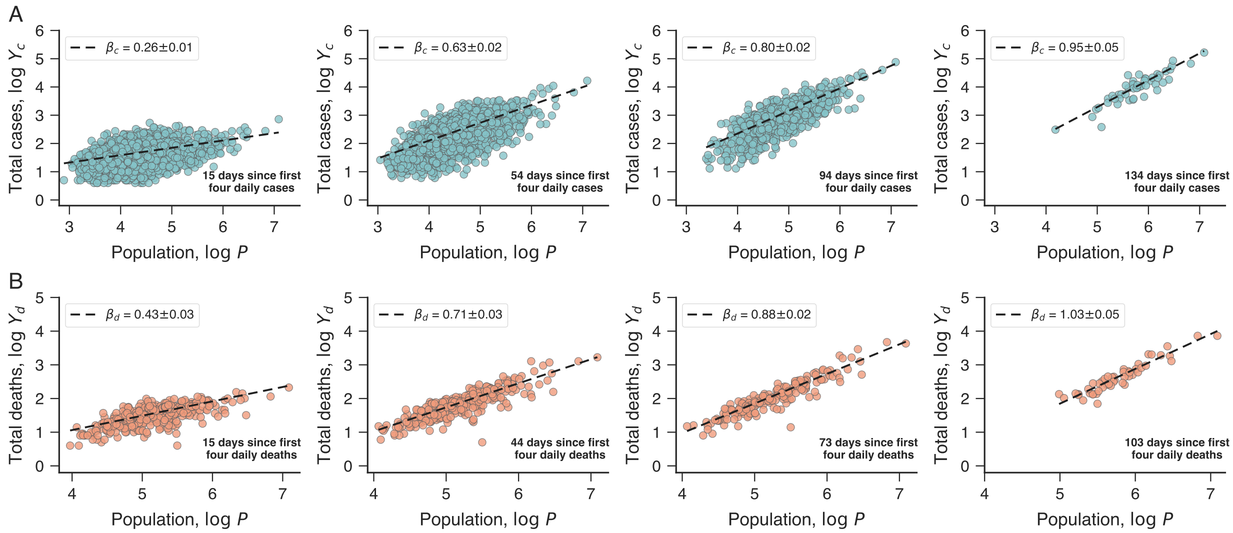

Figure 3. **Urban scaling relations of COVID-19 cases and deaths under different choices of values for the number of daily cases or daily deaths as reference points.** The same plots of Figure 1 in the main text but considering the first four daily cases and the first four daily deaths as reference points.

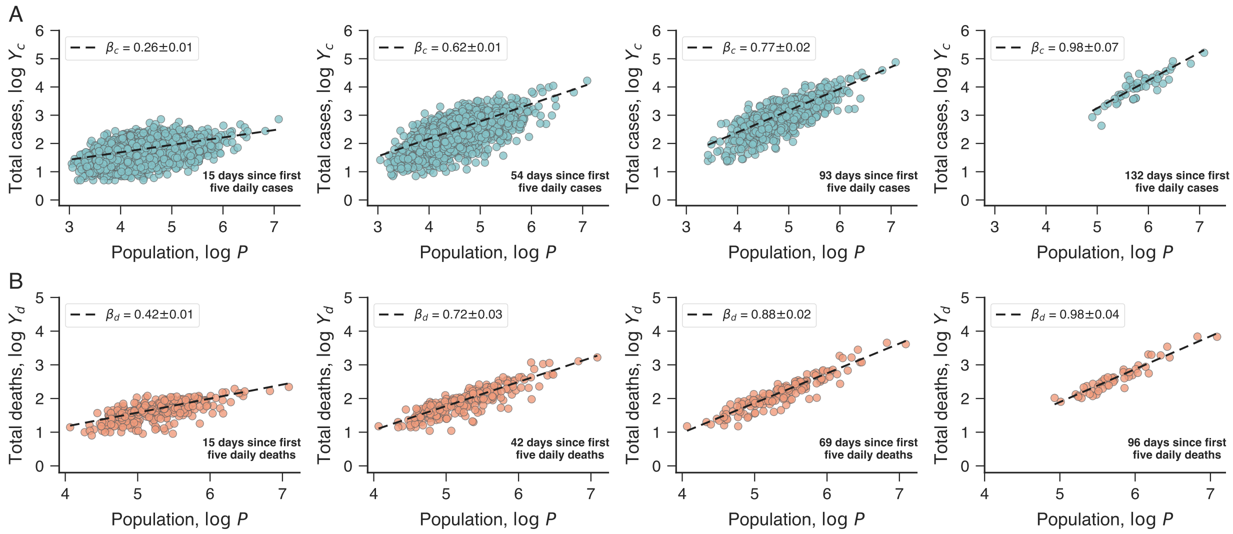

Figure 4. **Urban scaling relations of COVID-19 cases and deaths under different choices of values for the number of daily cases or daily deaths as reference points.** The same plots of Figure 1 in the main text but considering the first five daily cases and the first five daily deaths as reference points.

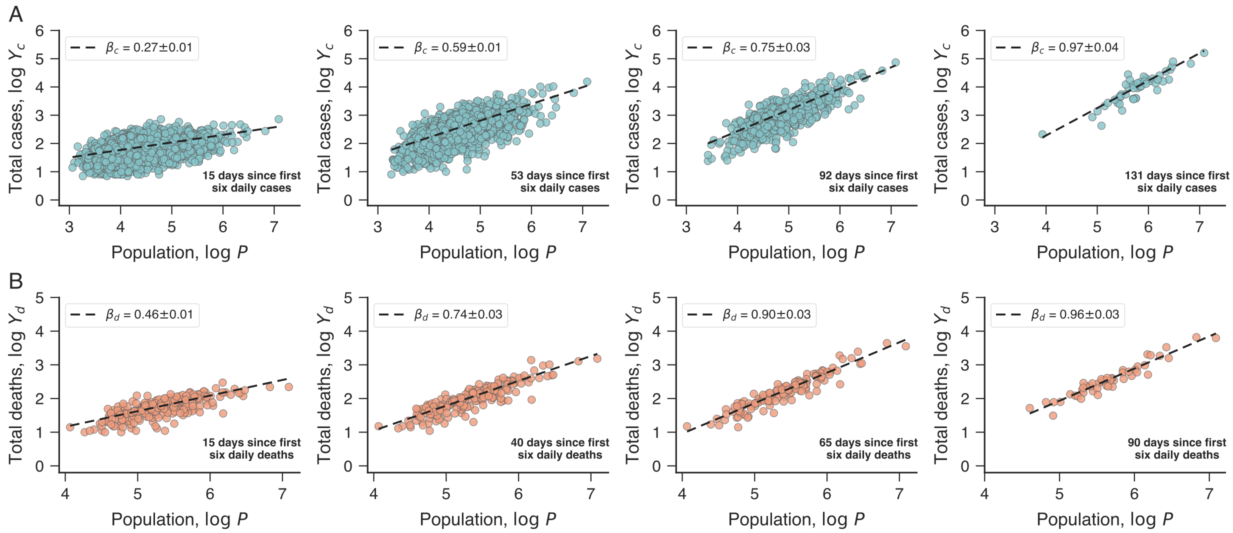

Figure 5. **Urban scaling relations of COVID-19 cases and deaths under different choices of values for the number of daily cases or daily deaths as reference points.** The same plots of Figure 1 in the main text but considering the first six daily cases and the first six daily deaths as reference points.

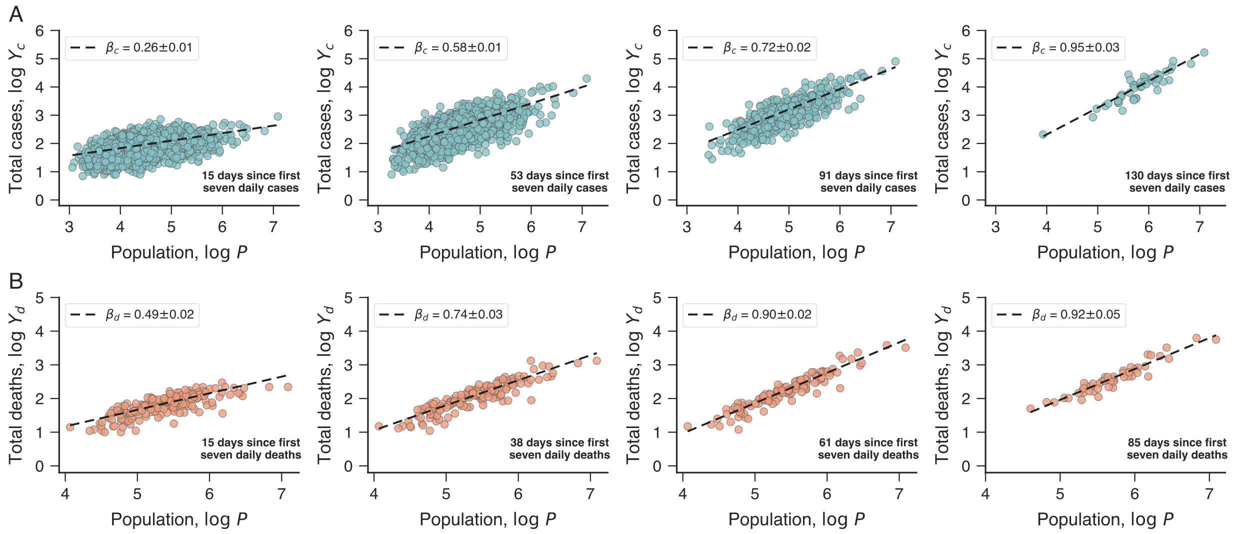

Figure 6. **Urban scaling relations of COVID-19 cases and deaths under different choices of values for the number of daily cases or daily deaths as reference points.** The same plots of Figure 1 in the main text but considering the first seven daily cases and the first seven daily deaths as reference points.

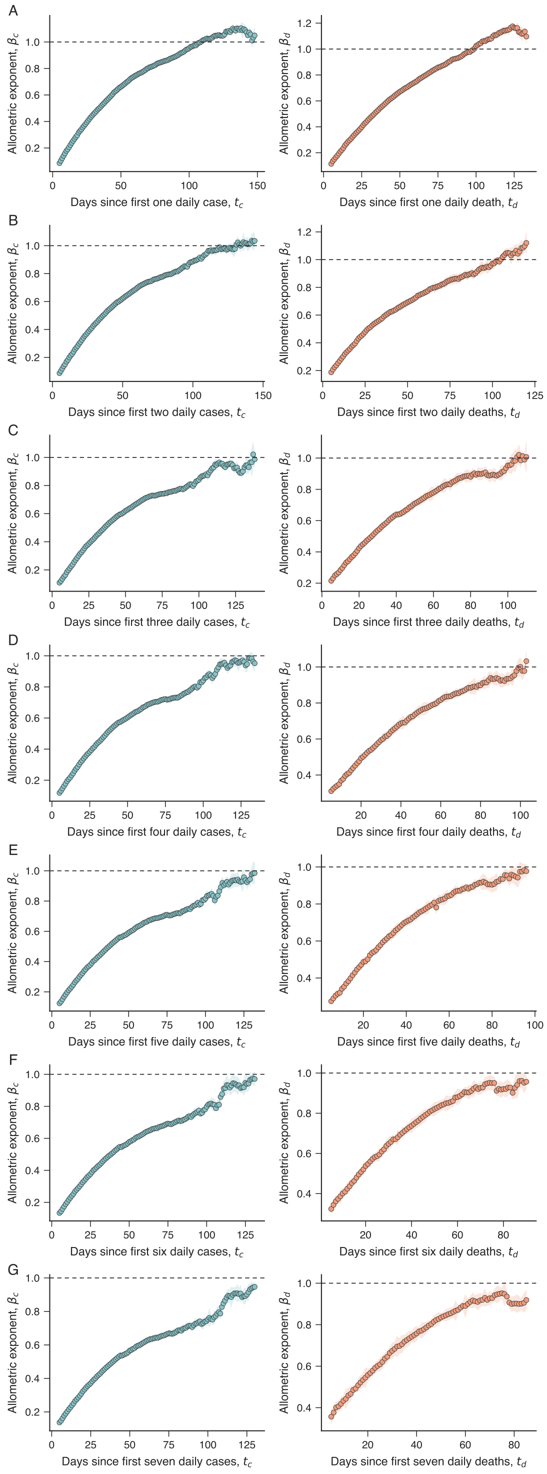

Figure 7. **Time dependence of the scaling exponents for COVID-19 cases and deaths under different choices of values for the number of daily cases or daily deaths as reference points.** Panels (A)-(G) show the dependence of the exponents β_c (left) and β_d (right) on t_c and t_d when considering the first 1-7 daily cases and the first 1-7 daily deaths as reference points. The shaded regions stand for standard errors, and the horizontal dashed lines represent $\beta_c = \beta_d = 1$. We note that the behavior observed for large numbers of the reference points appear to follow the behavior of small ones in the long-term course of the pandemic.

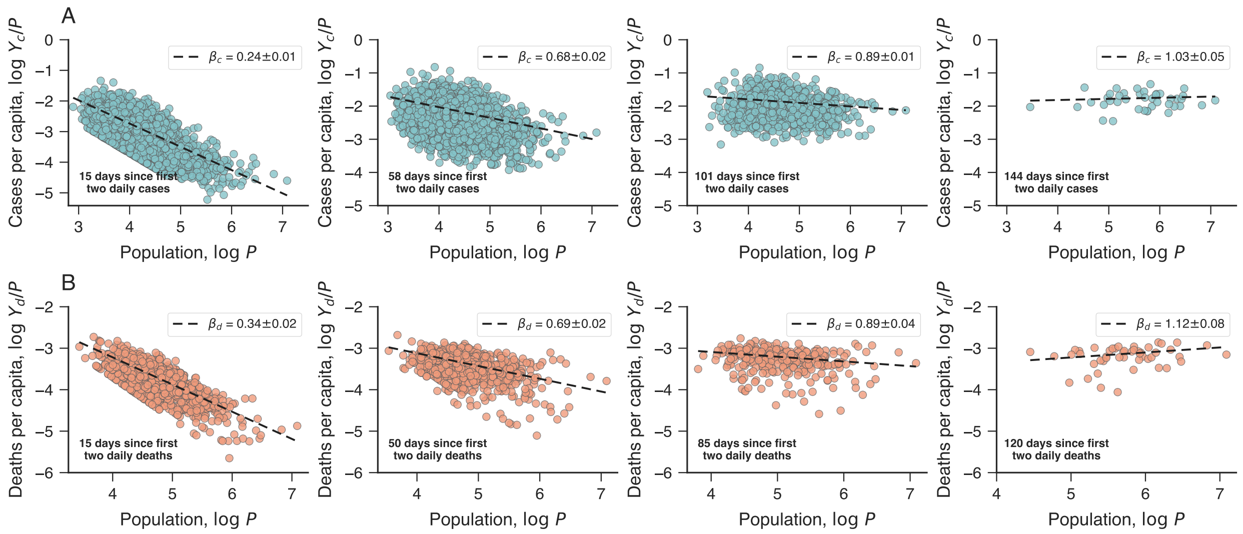

Figure 8. **Urban scaling relations of COVID-19 cases and deaths *per capita*.** (A) Relationship between the total of confirmed cases *per capita* of COVID-19 (Y_c/P) and city population (P) on logarithmic scale. (B) Relationship between the total of deaths *per capita* caused by COVID-19 (Y_d) and population (P) of Brazilian cities (on logarithmic scale). Panels show scaling relations on a particular day after the first two daily cases or the first two daily deaths. The dashed lines are the scaling relations with exponents indicated in each plot ($\beta_c - 1$ for cases *per capita* and $\beta_d - 1$ for deaths *per capita*).

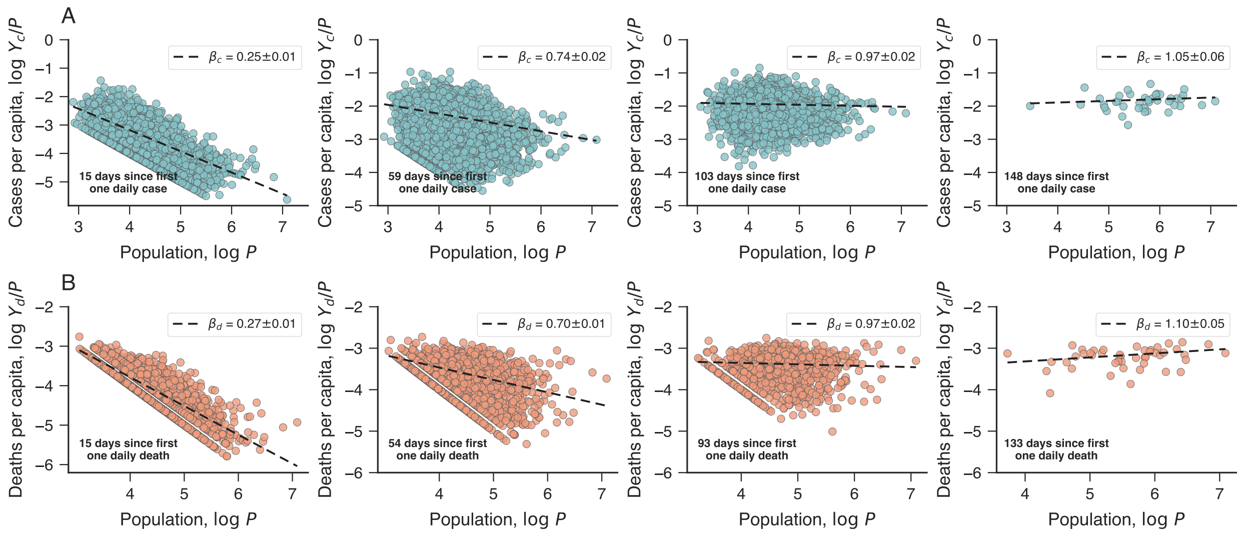

Figure 9. **Urban scaling relations of COVID-19 cases and deaths *per capita*.** The same as Figure 8 in this Appendix, but considering the first one daily case and the first one daily death as reference points.

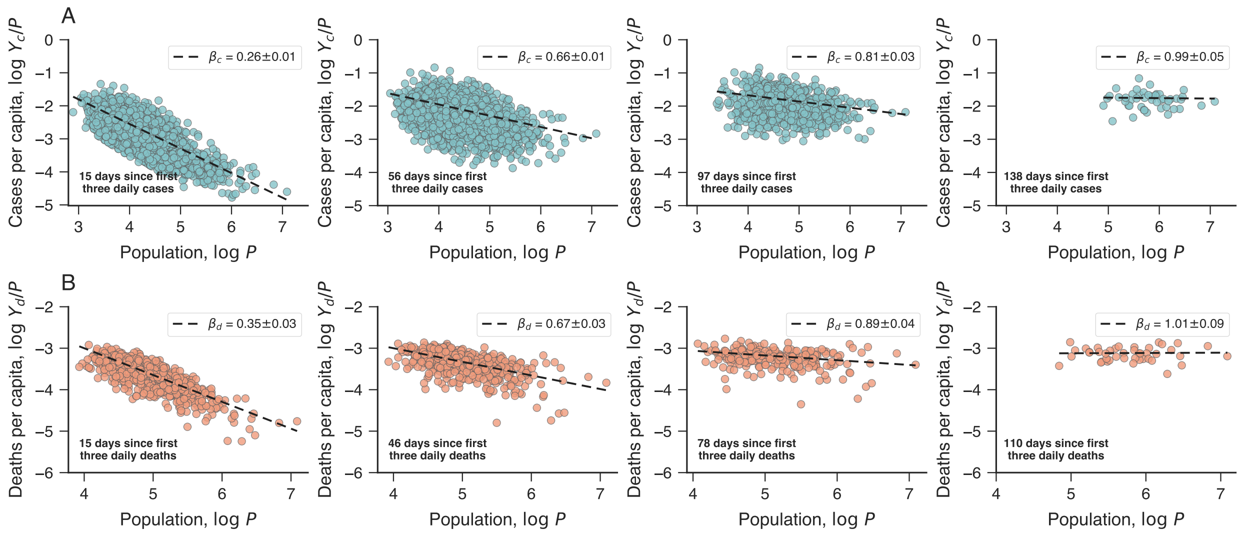

Figure 10. **Urban scaling relations of COVID-19 cases and deaths *per capita*.** The same as Figure 8 in this Appendix, but considering the first three daily cases and the first three daily deaths as reference points.

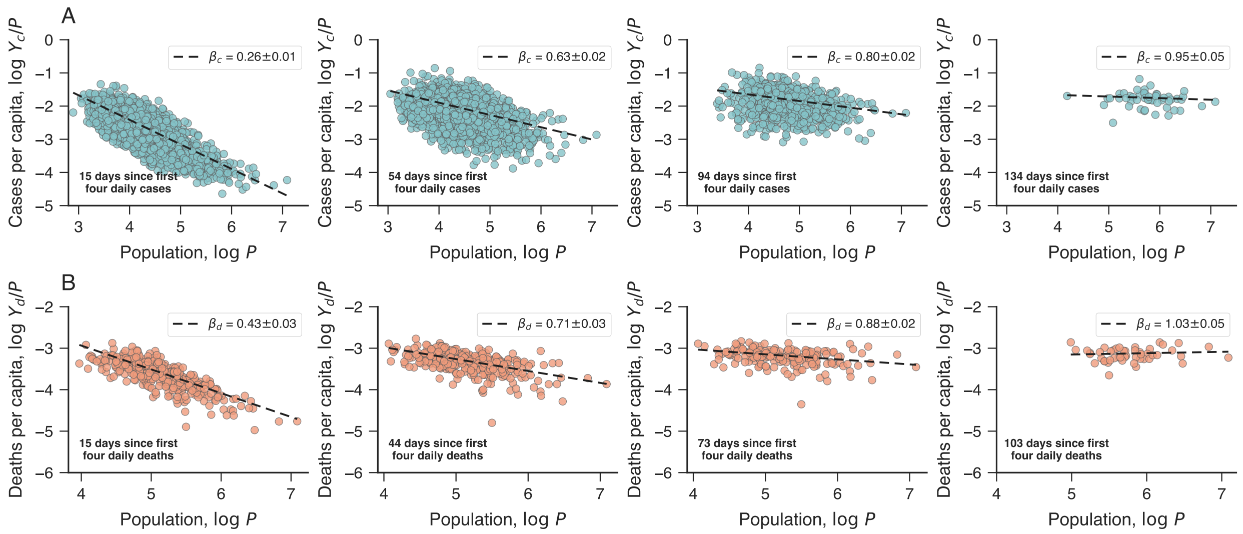

Figure 11. **Urban scaling relations of COVID-19 cases and deaths *per capita*.** The same as Figure 8 in this Appendix, but considering the first four daily cases and the first four daily deaths as reference points.

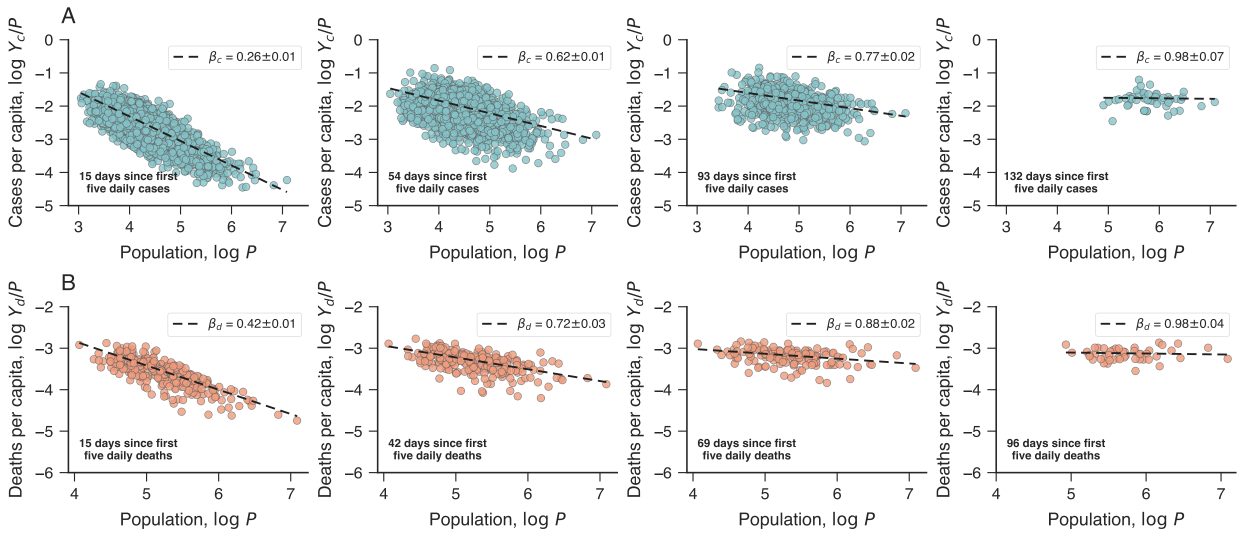

Figure 12. **Urban scaling relations of COVID-19 cases and deaths *per capita*.** The same as Figure 8 in this Appendix, but considering the first five cases and the first five daily deaths as reference points.

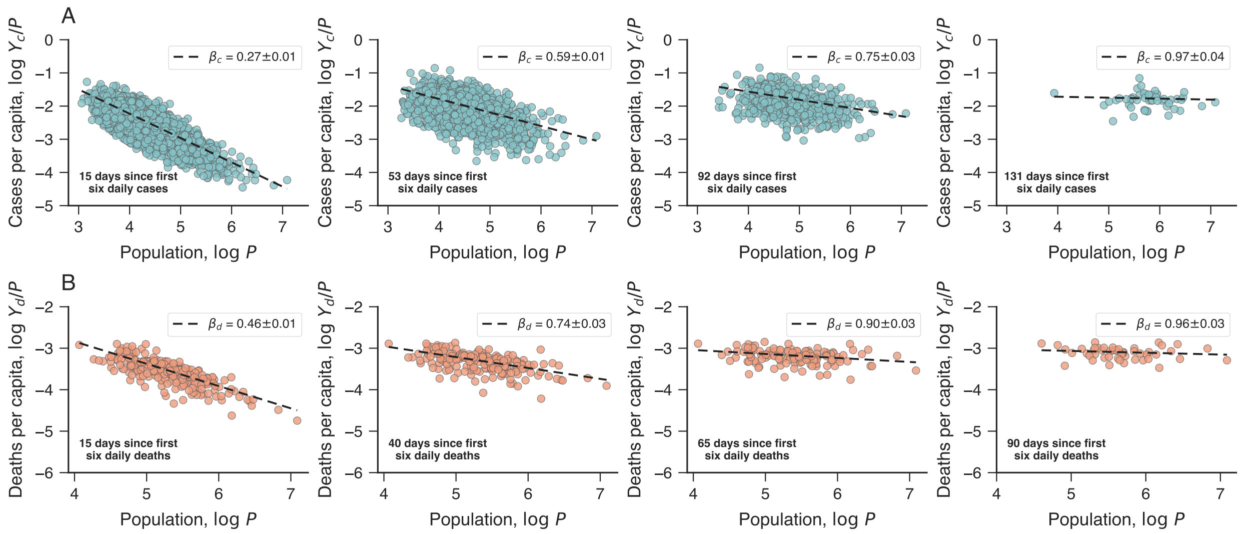

Figure 13. **Urban scaling relations of COVID-19 cases and deaths *per capita*.** The same as Figure 8 in this Appendix, but considering the first six daily cases and the first six daily deaths as reference points.

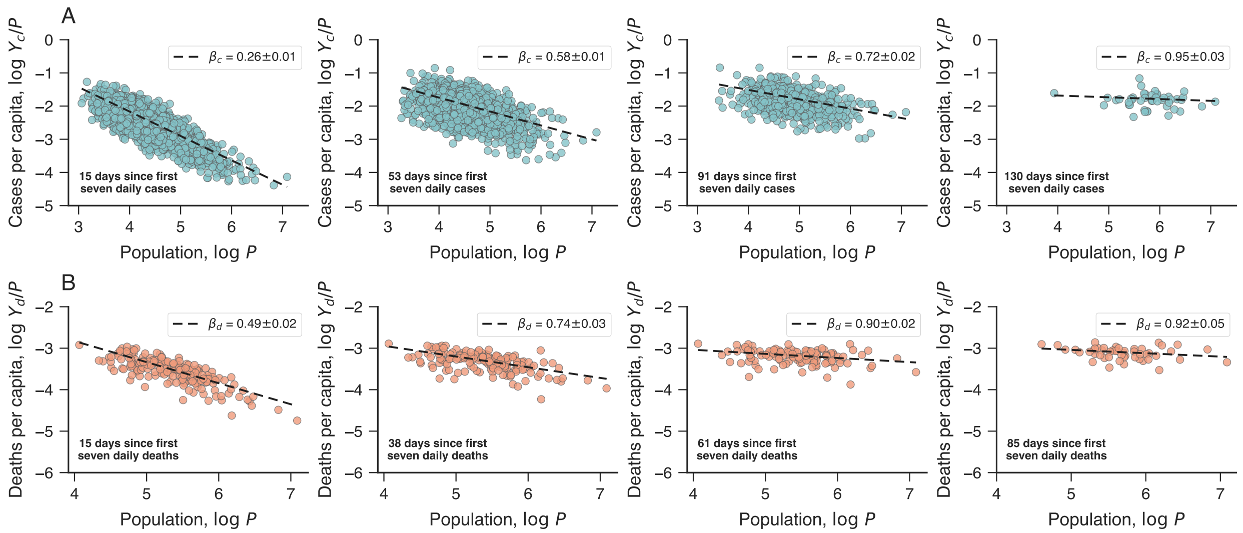

Figure 14. **Urban scaling relations of COVID-19 cases and deaths *per capita*.** The same as Figure 8 in this Appendix, but considering the first seven daily cases and the first seven daily deaths as reference points.

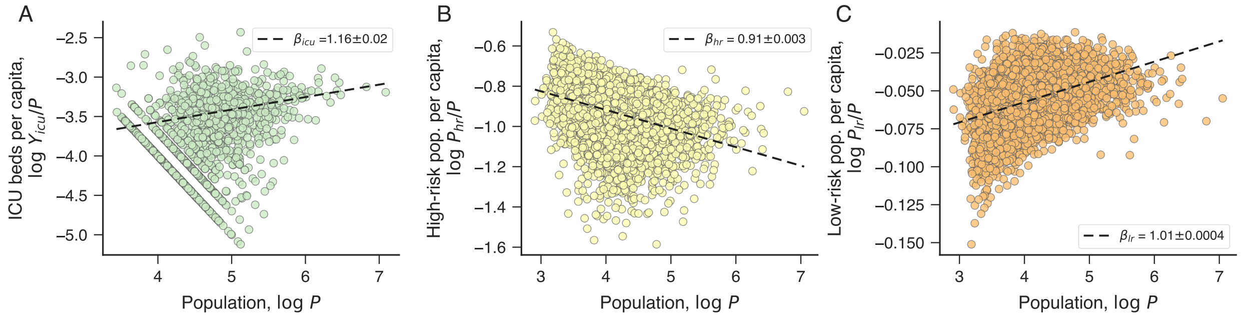

Figure 15. **Urban scaling of ICU beds, high-risk and low-risk populations *per capita*.** (A) Relationship between the number of ICU beds *per capita* (Y_{icu}/P) and the city population (P) on logarithmic scale. (B) Relationship between the high-risk population *per capita* (P_{hr}/P) and the city population (P) on logarithmic scale. (C) Relationship between low-risk population *per capita* (P_{lr}/P) and the city population (P) on logarithmic scale. In all panels, the dashed lines are the scaling relations with exponents indicated in each plot ($\beta_{icu}-1$ for ICU beds *per capita*, $\beta_{hr}-1$ for high-risk population *per capita*, and $\beta_{lr}-1$ for low-risk population *per capita*).

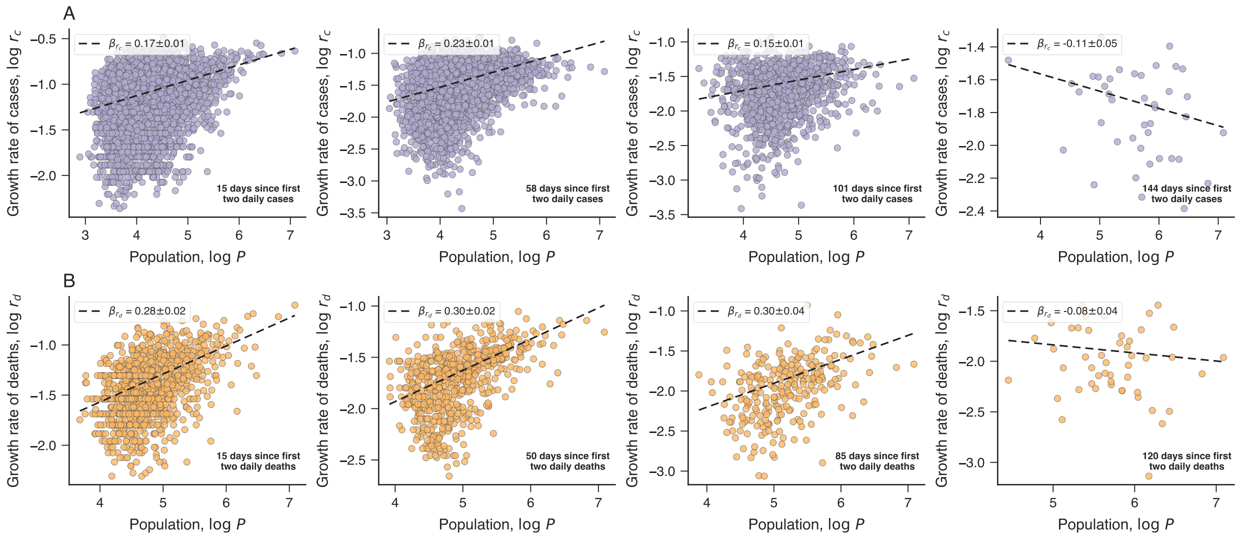

Figure 16. **Urban scaling relations of growth rates of COVID-19 cases and deaths.** (A) Relationship between the growth rate of cases (r_c) and the city population (P) on logarithmic scale. Panels show scaling relations for values of r_c estimated after a given number of days since the first two daily cases (four evenly spaced values of t_c between 15 days and the largest value yielding at least 50 cities, as indicated within panels). (B) Relationship between the growth rate of deaths (r_d) and the city population (P) on logarithmic scale. Panels show scaling relations for values of r_d estimated after a given number of days since the first two daily deaths (four evenly spaced values of t_d between 15 days and the largest value yielding at least 50 cities, as indicated within panels). The markers in (A) and (B) represent cities, and the dashed lines are the adjusted scaling relations with best-fitting exponents indicated in each plot (β_{r_c} for the growth rate of cases and β_{r_d} for the growth rate of deaths). All rates were estimated using $\tau = 14$ as defined in Eq. (5) of the main text.

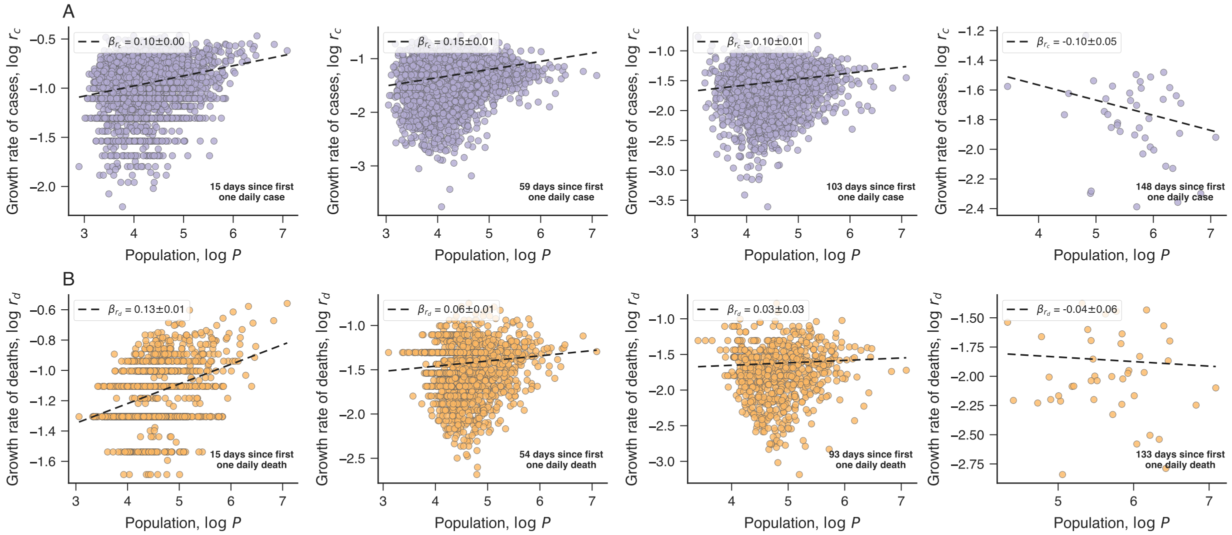

Figure 17. **Urban scaling relations of growth rates of COVID-19 cases and deaths.** The same as Figure 16 in this Appendix, but considering the first one daily case and first one daily death as reference points.

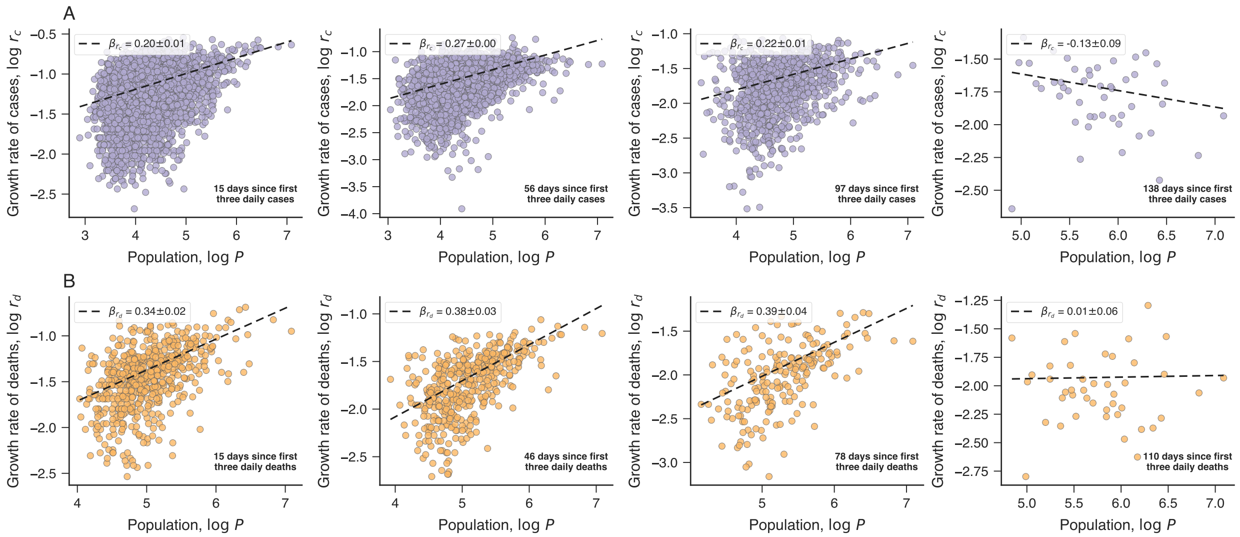

Figure 18. **Urban scaling relations of growth rates of COVID-19 cases and deaths.** The same as Figure 16 in this Appendix, but considering three daily cases and three daily deaths as reference points.

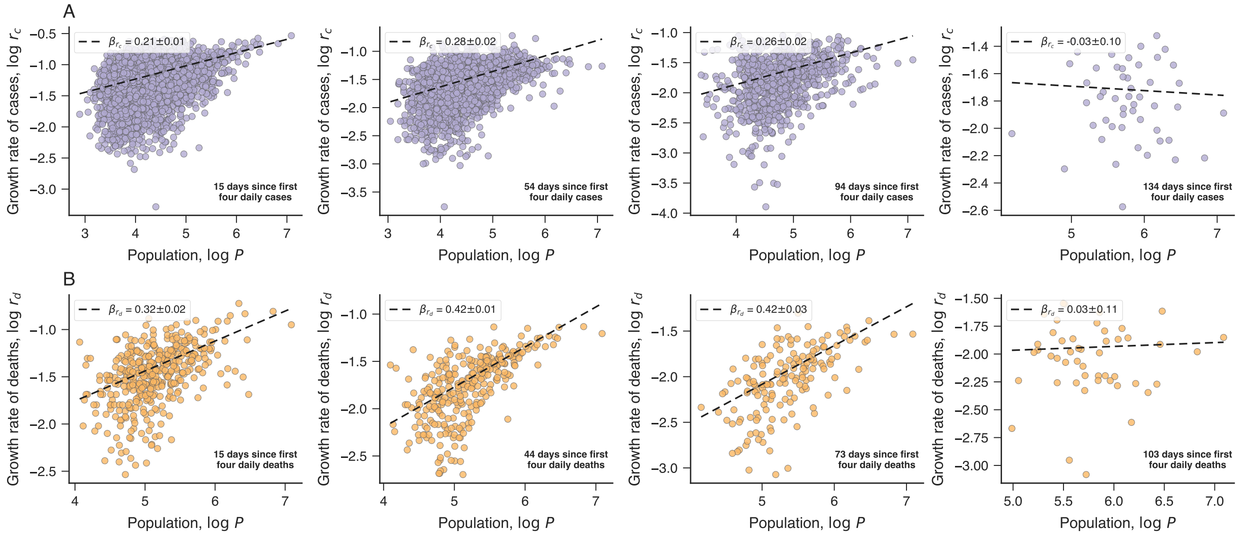

Figure 19. **Urban scaling relations of growth rates of COVID-19 cases and deaths.** The same as Figure 16 in this Appendix, but considering four daily cases and four daily deaths as reference points.

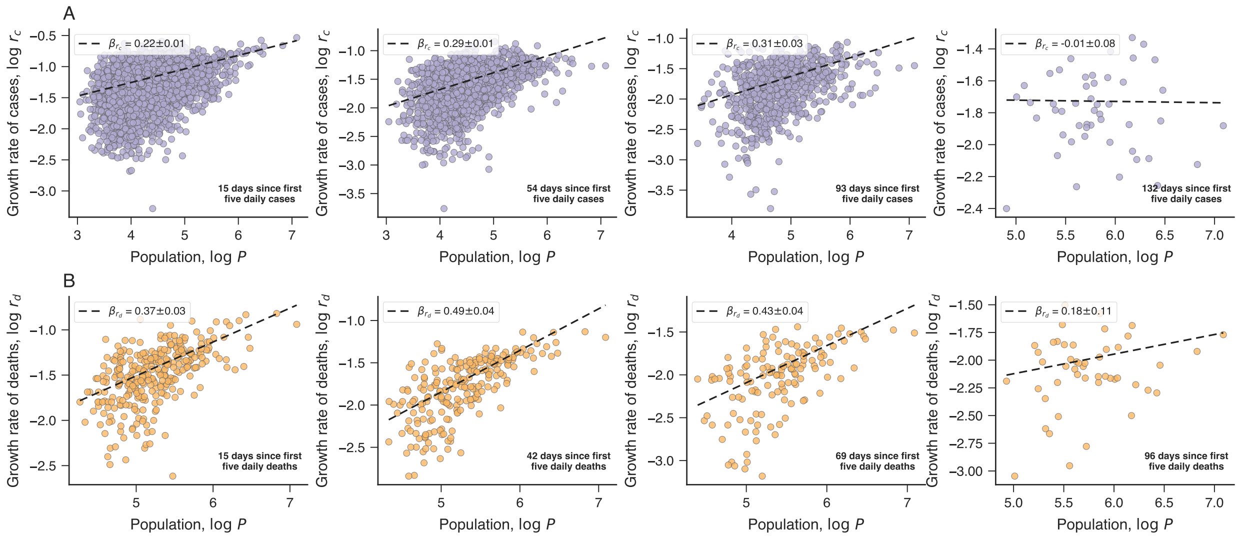

Figure 20. **Urban scaling relations of growth rates of COVID-19 cases and deaths.** The same as Figure 16 in this Appendix, but considering five daily cases and five daily deaths as reference points.

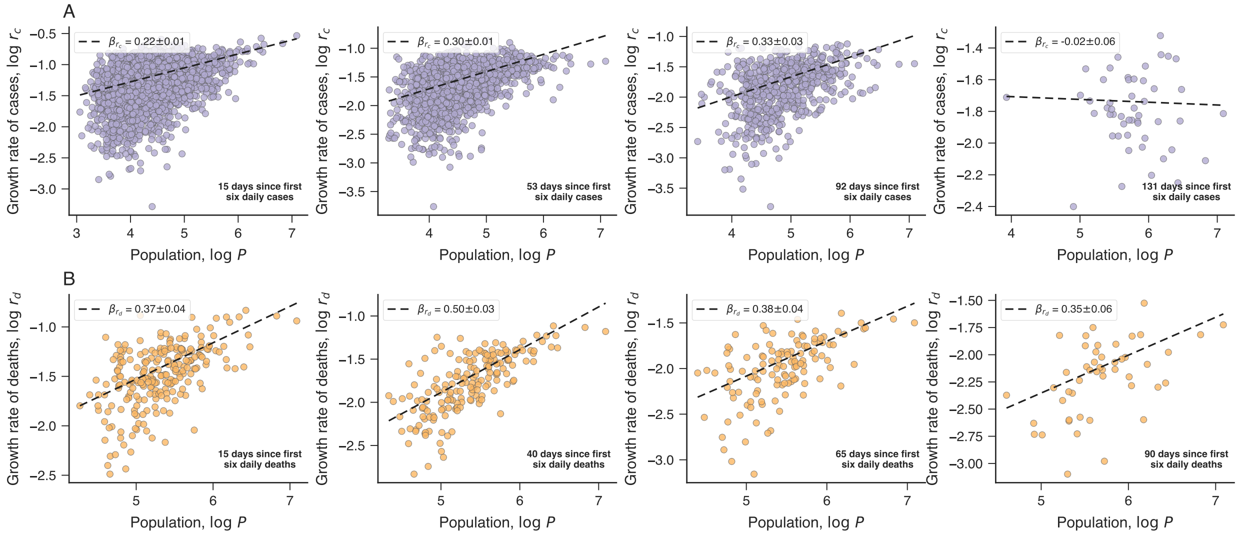

Figure 21. **Urban scaling relations of growth rates of COVID-19 cases and deaths.** The same as Figure 16 in this Appendix, but considering six daily cases and six daily deaths as reference points.

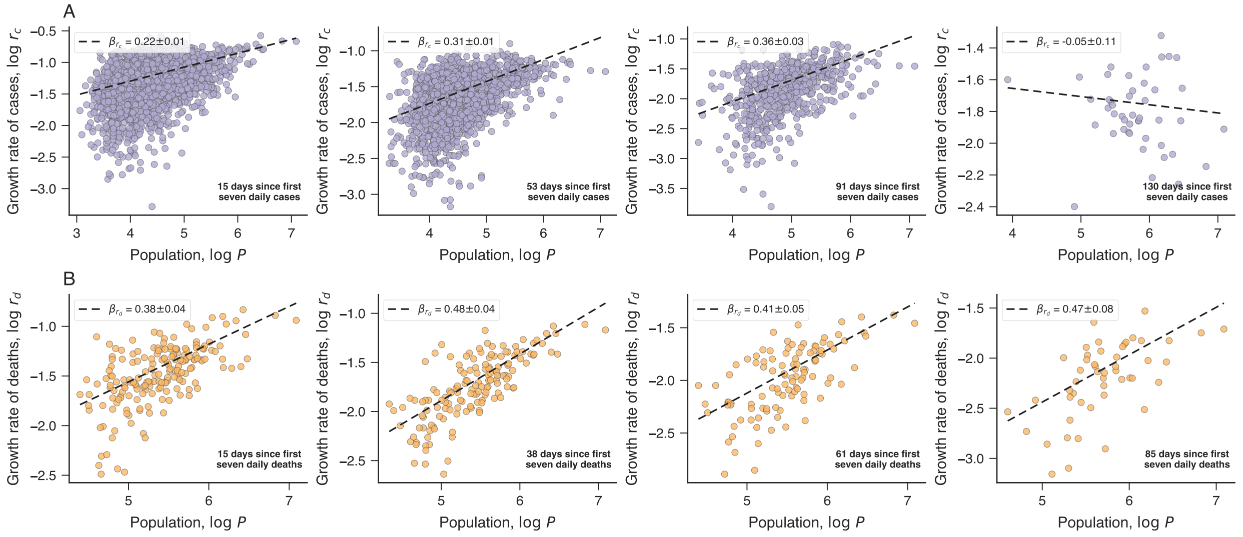

Figure 22. **Urban scaling relations of growth rates of COVID-19 cases and deaths.** The same as Figure 16 in this Appendix, but considering seven daily cases and seven daily deaths as reference points.

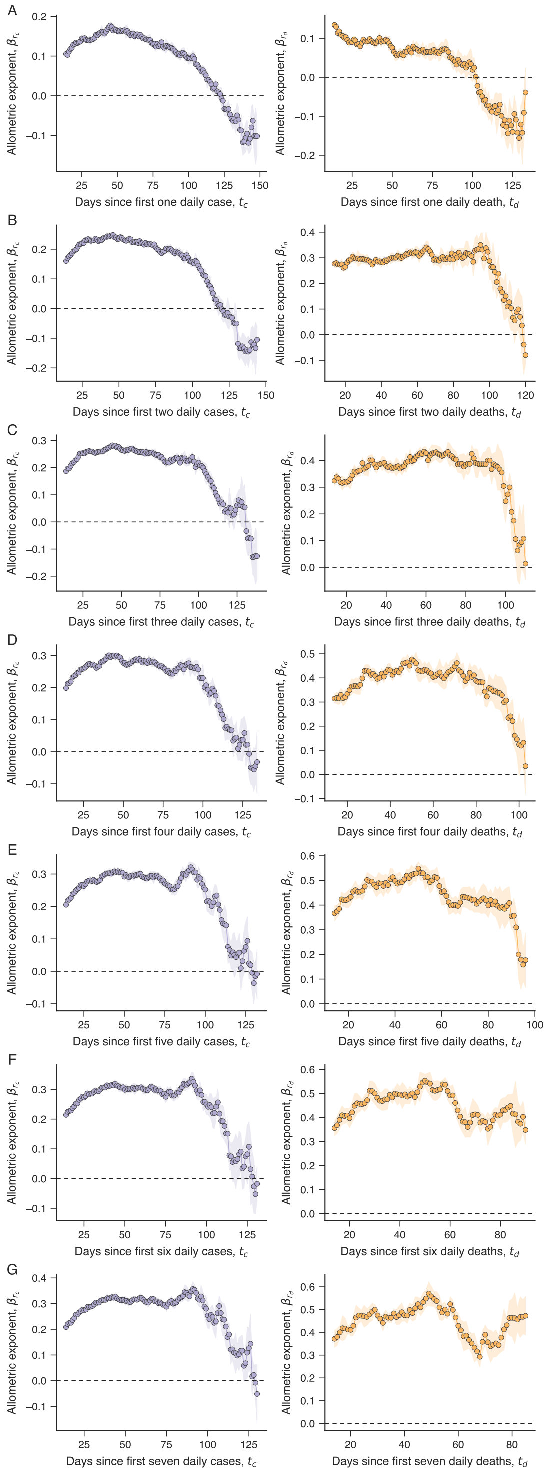

Figure 23. **Time dependence of the scaling exponents for growth rates of cases and deaths under different choices for the number of daily cases or daily deaths as reference points.** Panels (A)-(G) show the dependence of the exponents β_{r_c} and β_{r_d} on t_c and t_d when considering the first 1-7 daily cases and the first 1-7 daily deaths as reference points. The shaded regions stand for standard errors, and the horizontal dashed lines represent $\beta_{r_c} = \beta_{r_d} = 0$. We notice that behavior observed for large numbers of the reference points appear to follow the behavior of small ones in the long-term course of the pandemic.

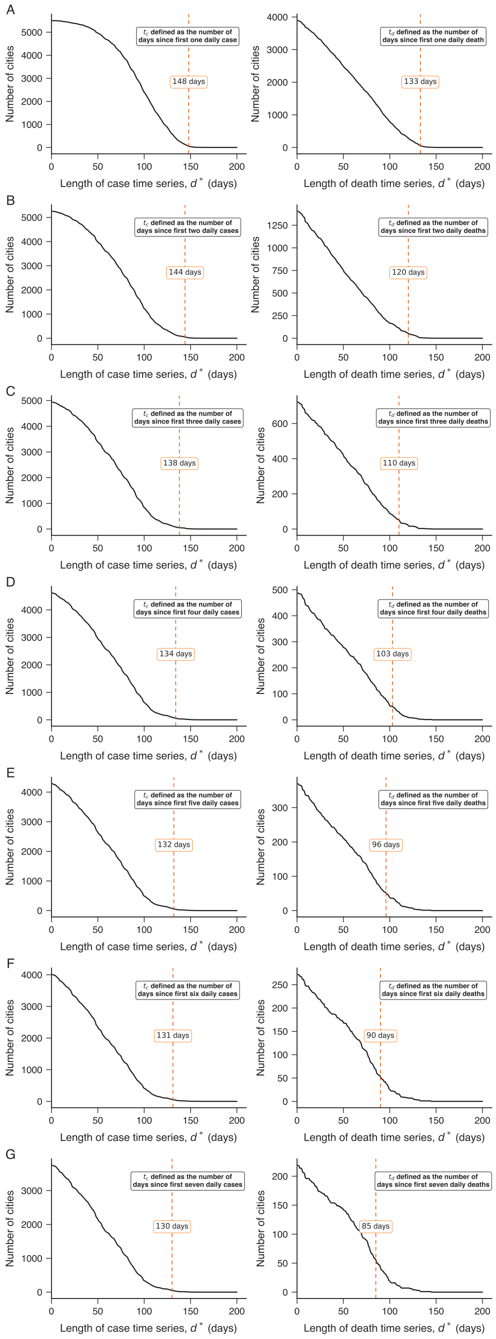

Figure 24. **Number of cities with time series longer than a particular number of days.** Left panels show the number of cities reporting cases of COVID-19 with time series larger than d^* days. The vertical dashed lines indicate that there are 50 cities with confirmed cases time series longer than the particular of number of days indicated within the plots. Right panels show the number of cities reporting deaths caused by COVID-19 with time series longer than d^* days. The vertical dashed lines indicate that there are 50 cities with deaths time series longer than the particular of number of days indicated within the plots. We have used these thresholds to ensure that our scaling relations are estimated from samples sizes having at least 50 cities. Panels (A)-(G) show the results when considering the first 1-7 daily cases and the first 1-7 daily deaths as reference points.

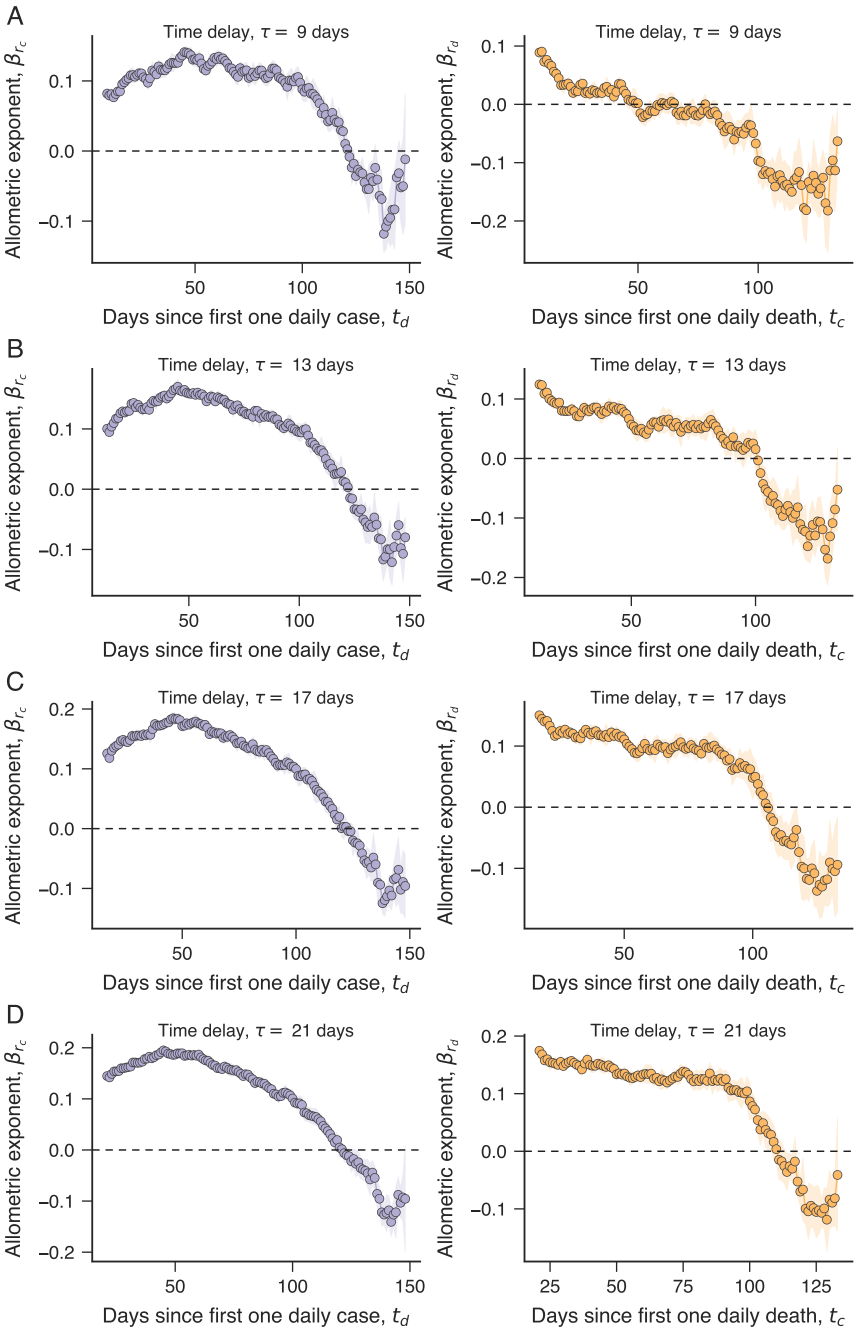

Figure 25. **Variations in the scaling exponents for the growth rates of cases and deaths under different values of time delay τ.** Panels (A)-(D) show the dependence of the exponent β_{r_c} (left panels) and β_{r_d} (right panels) on the number of days since the first one daily case (t_c) or death (t_d) for different values of τ.

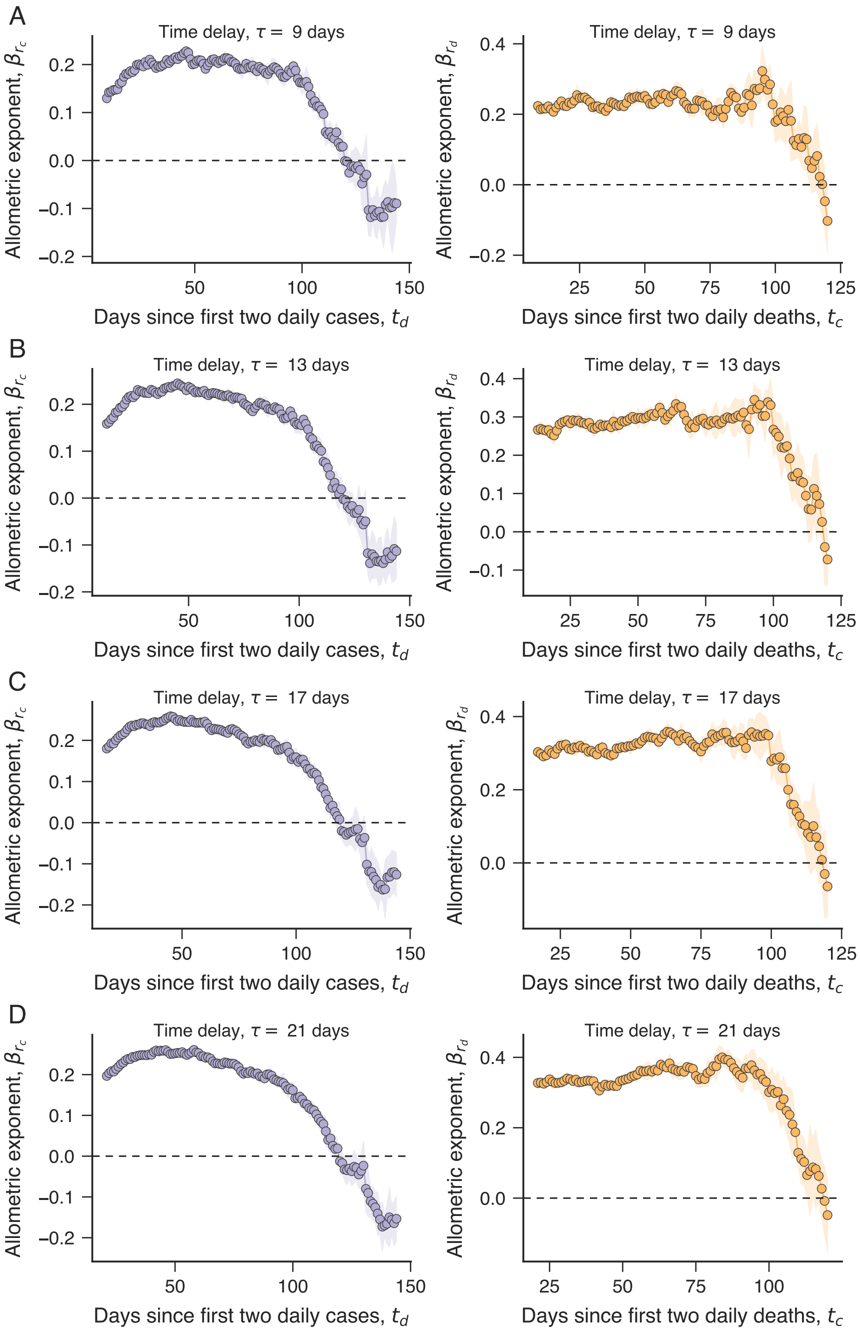

Figure 26. **Variations in the scaling exponents for the growth rates of cases and deaths under different values of time delay τ.** Panels (A)-(D) show the dependence of the exponent β_{r_c} (left panels) and β_{r_d} (right panels) on the number of days since the first two daily cases (t_c) or deaths (t_d) for different values of τ.

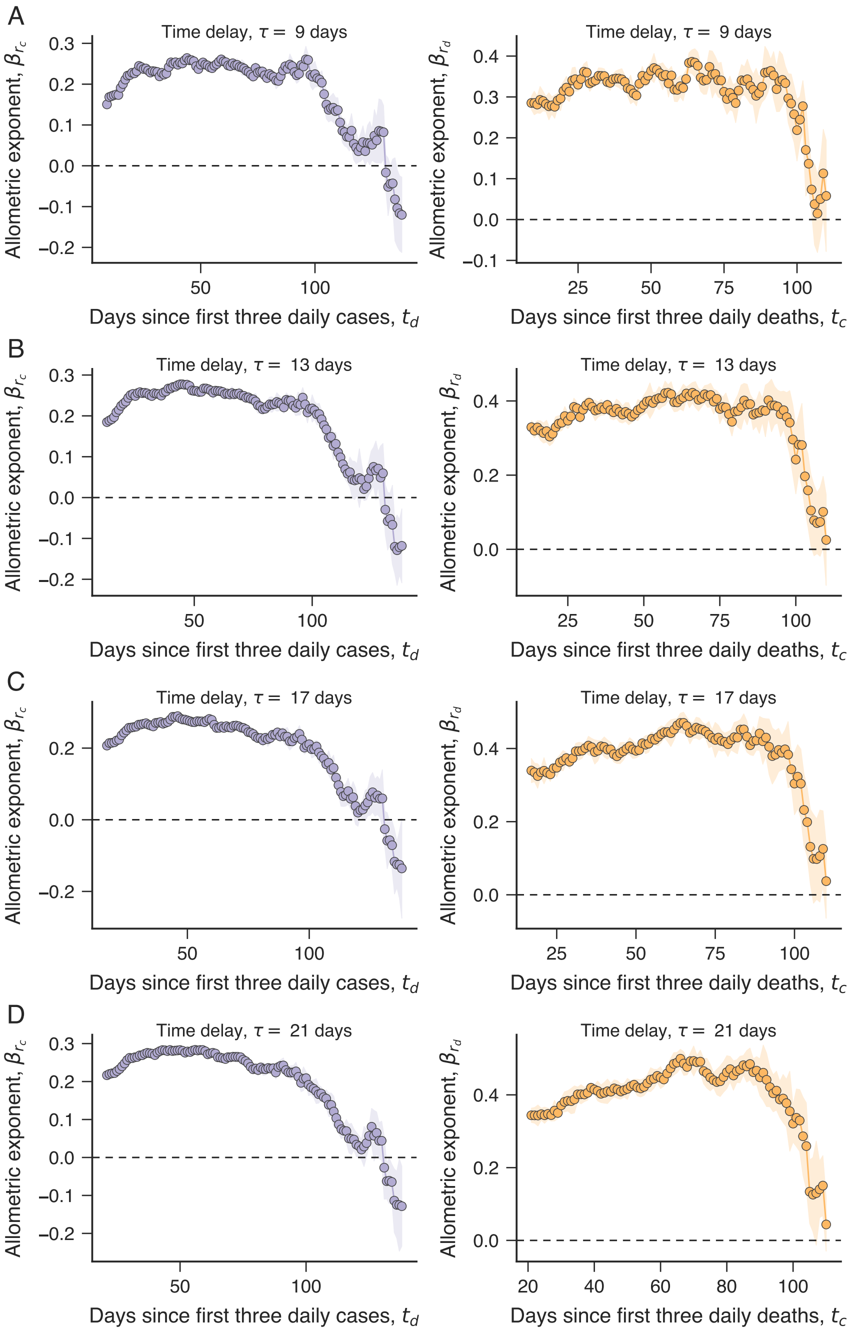

Figure 27. **Variations in the scaling exponents for the growth rates of cases and deaths under different values of time delay τ.** Panels (A)-(D) show the dependence of the exponent β_{r_c} (left panels) and β_{r_d} (right panels) on the number of days since the first three daily cases (t_c) or deaths (t_d) for different values of τ.

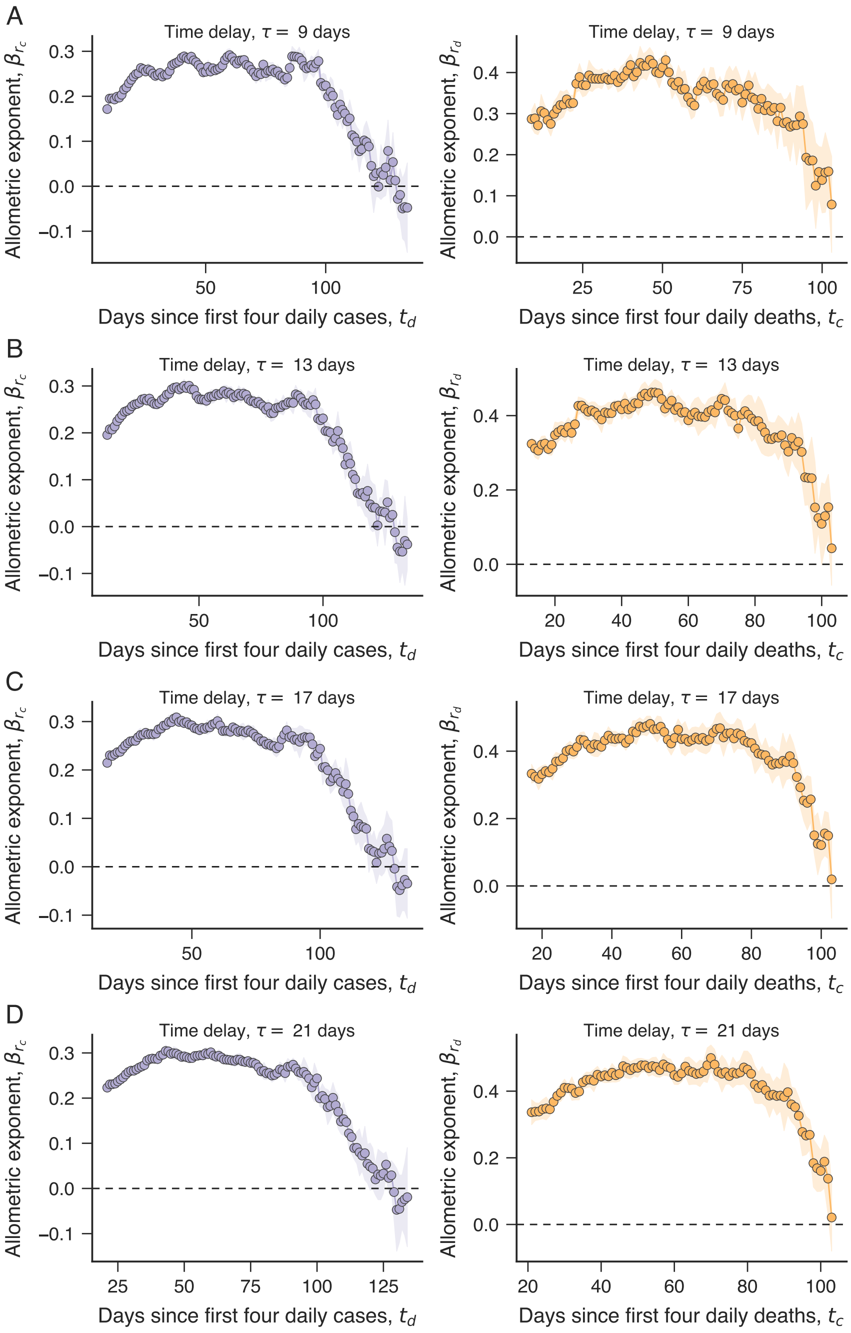

Figure 28. **Variations in the scaling exponents for the growth rates of cases and deaths under different values of time delay τ.** Panels (A)-(D) show the dependence of the exponent β_{r_c} (left panels) and β_{r_d} (right panels) on the number of days since the first four daily cases (t_c) or deaths (t_d) for different values of τ.

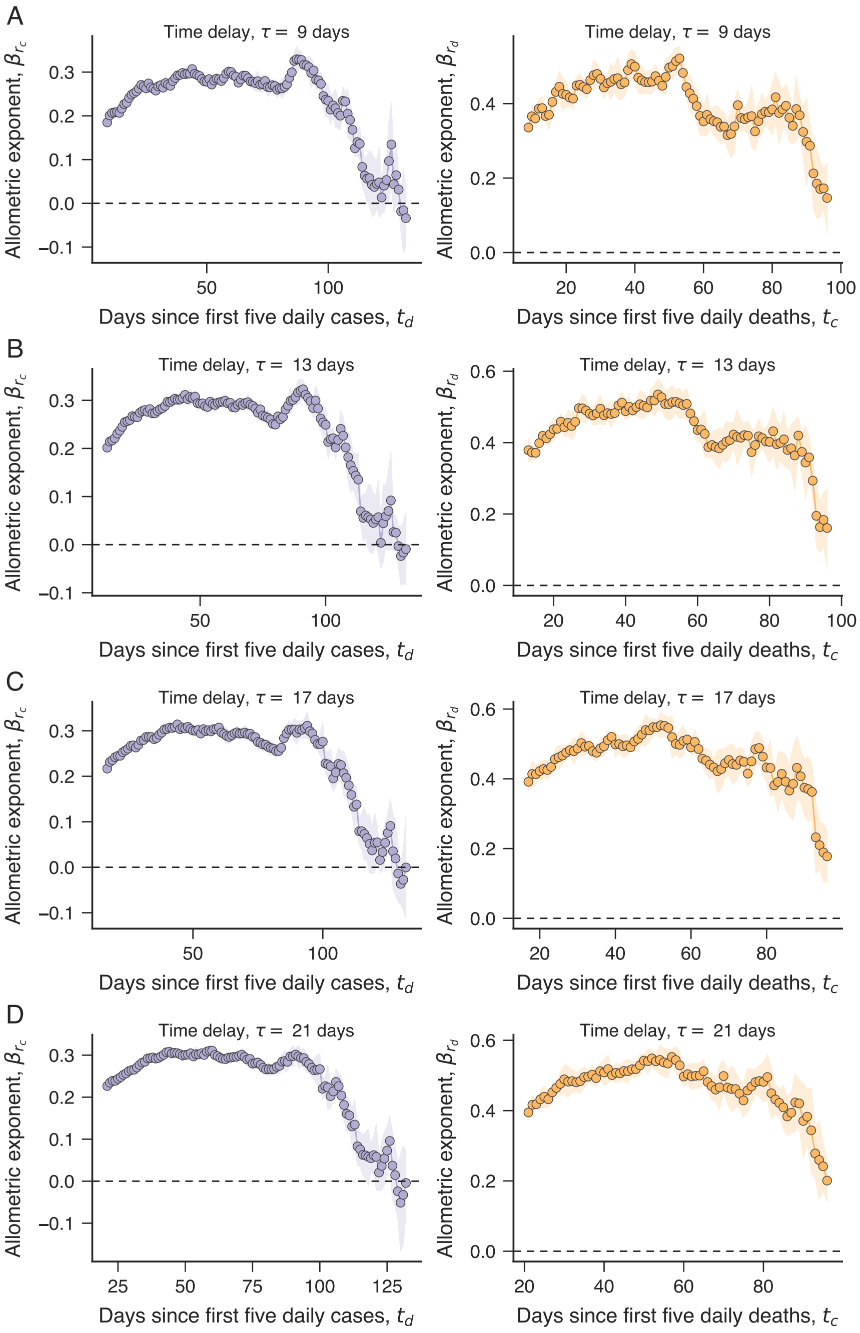

Figure 29. **Variations in the scaling exponents for the growth rates of cases and deaths under different values of time delay τ.** Panels (A)-(D) show the dependence of the exponent β_{r_c} (left panels) and β_{r_d} (right panels) on the number of days since the first five daily cases (t_c) or deaths (t_d) for different values of τ.

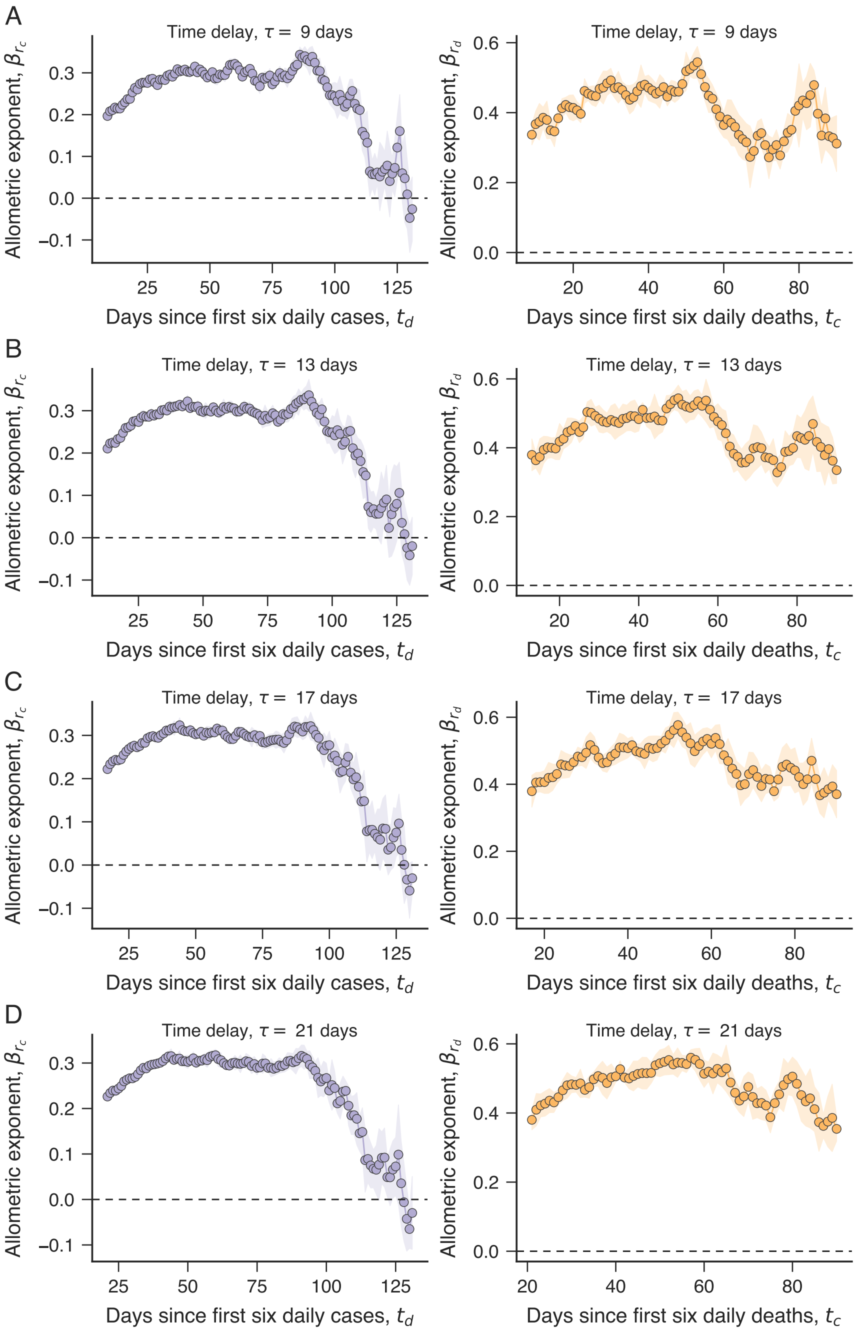

Figure 30. **Variations in the scaling exponents for the growth rates of cases and deaths under different values of time delay τ.** Panels (A)-(D) show the dependence of the exponent β_{r_c} (left panels) and β_{r_d} (right panels) on the number of days since the first six daily cases (t_c) or deaths (t_d) for different values of τ.

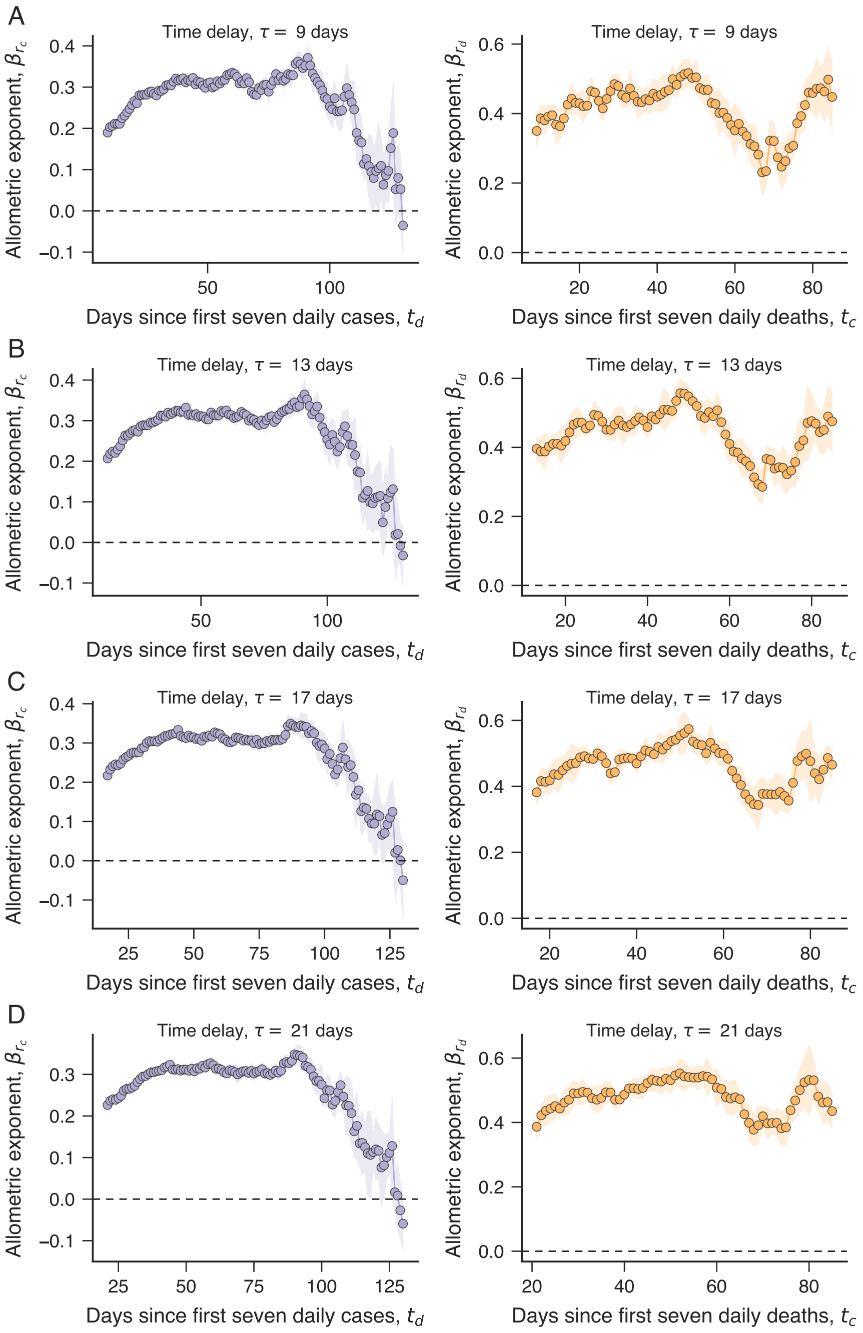

Figure 31. **Variations in the scaling exponents for the growth rates of cases and deaths under different values of time delay τ.** Panels (A)-(D) show the dependence of the exponent β_{r_c} (left panels) and β_{r_d} (right panels) on the number of days since the first seven daily cases (t_c) or deaths (t_d) for different values of τ.

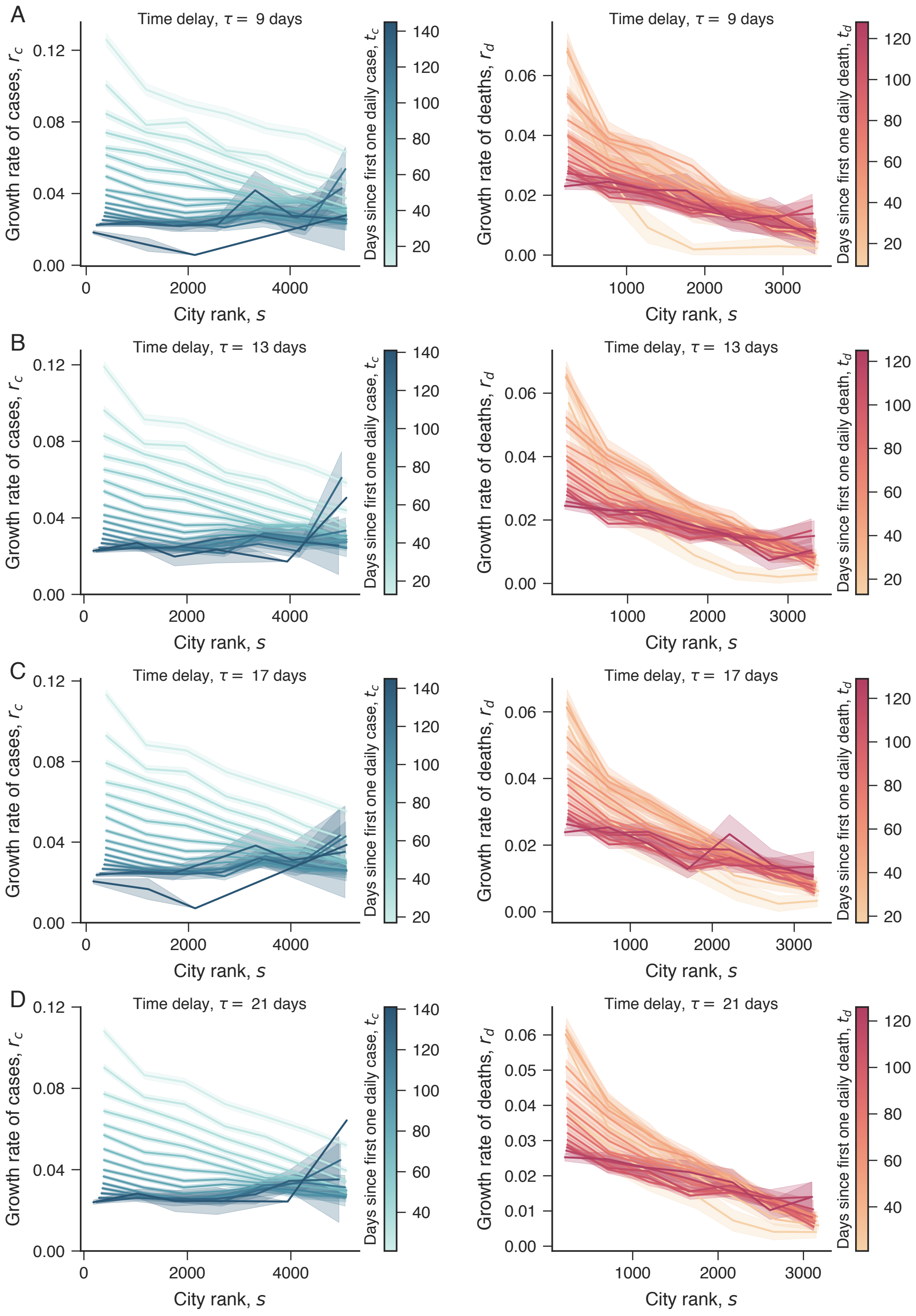

Figure 32. **Variations in the association between the growth rates and the city rank under different values of time delay τ.** Panels (A)-(D) show the average relationship between the growth rates of COVID-19 cases (r_c, left panels) and deaths (r_d, right panels) and the city rank s for number of days since the first one daily case (t_c) or death (t_d) and for different values of τ (indicated within the plots).

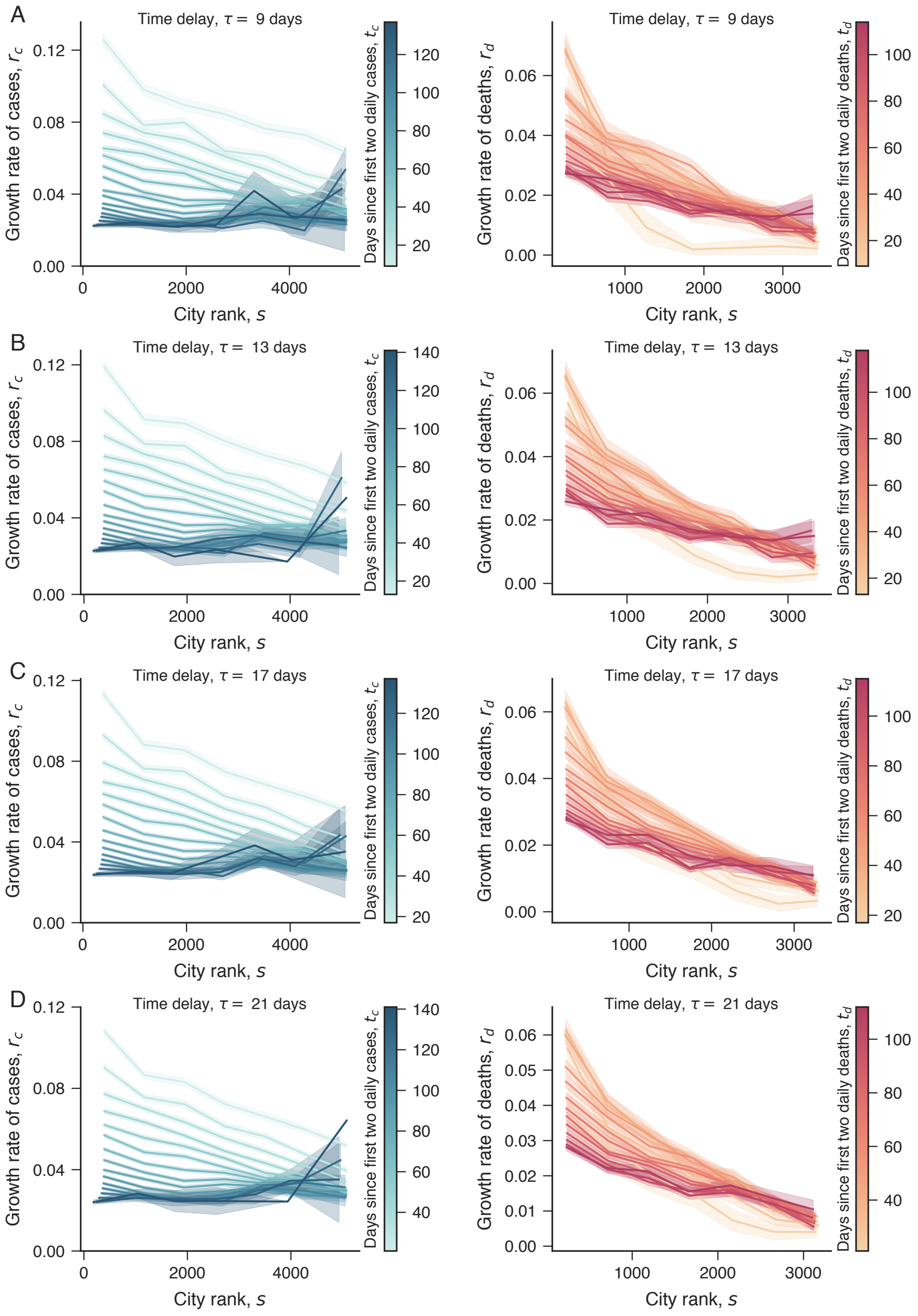

Figure 33. **Variations in the association between the growth rates and the city rank under different values of time delay τ.** Panels (A)-(D) show the average relationship between the growth rates of COVID-19 cases (r_c, left panels) and deaths (r_d, right panels) and the city rank s for number of days since the first two daily cases (t_c) or deaths (t_d) and for different values of τ (indicated within the plots).

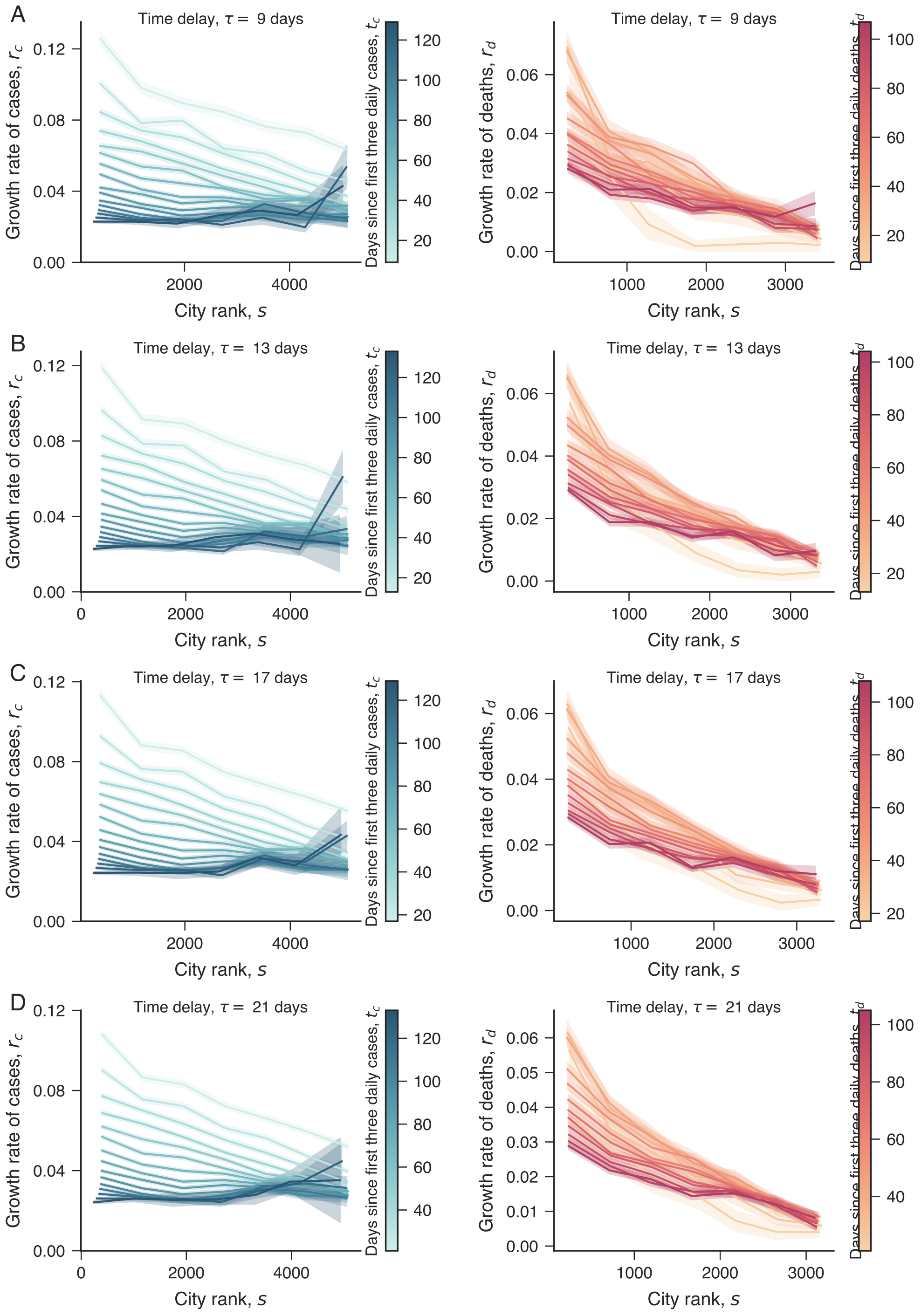

Figure 34. **Variations in the association between the growth rates and the city rank under different values of time delay τ.** Panels (A)-(D) show the average relationship between the growth rates of COVID-19 cases (r_c, left panels) and deaths (r_d, right panels) and the city rank s for number of days since the first three daily cases (t_c) or deaths (t_d) and for different values of τ (indicated within the plots).

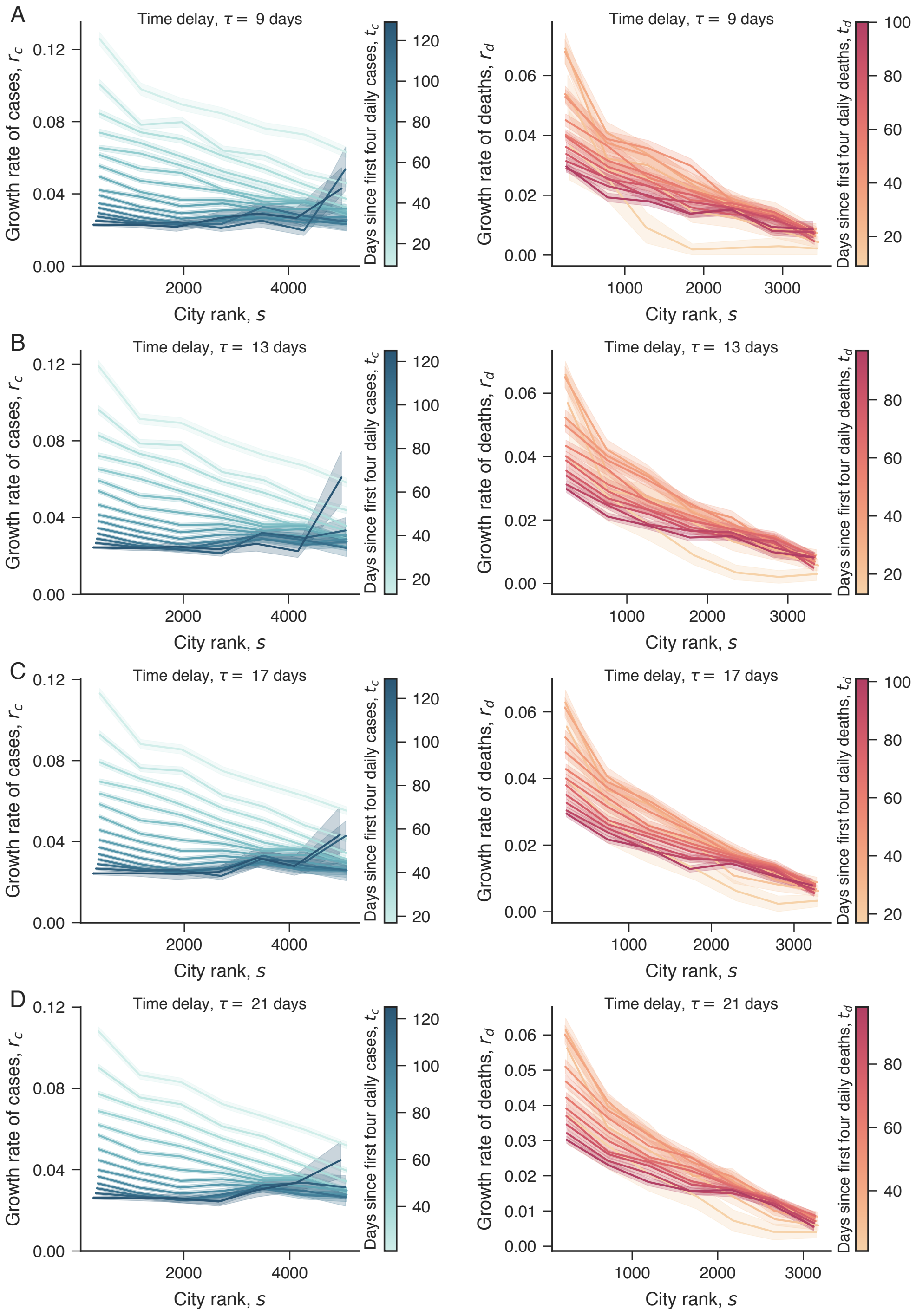

Figure 35. **Variations in the association between the growth rates and the city rank under different values of time delay τ.** Panels (A)-(D) show the average relationship between the growth rates of COVID-19 cases (r_c, left panels) and deaths (r_d, right panels) and the city rank s for number of days since the first four daily cases (t_c) or deaths (t_d) and for different values of τ (indicated within the plots).

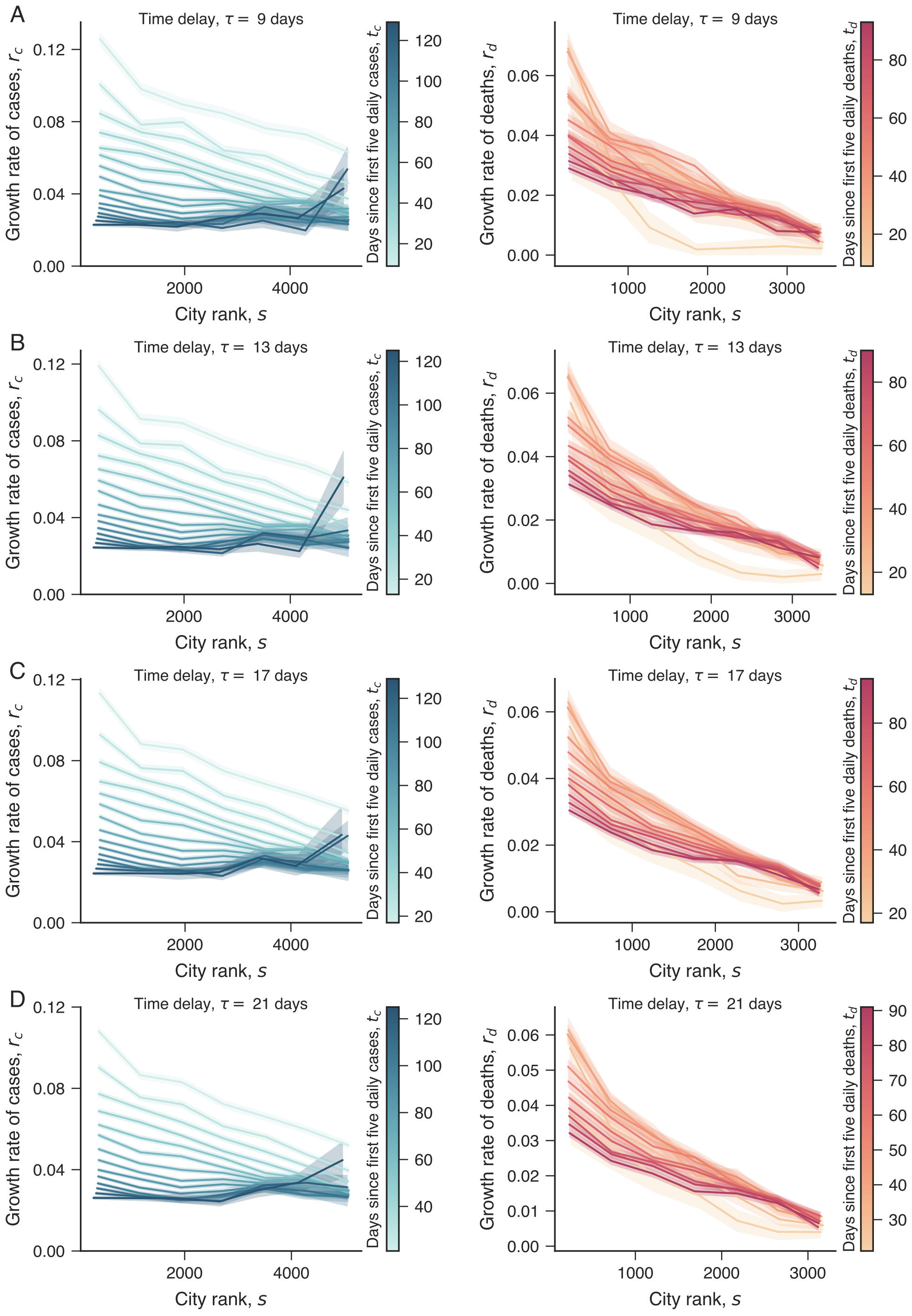

Figure 36. **Variations in the association between the growth rates and the city rank under different values of time delay τ.** Panels (A)-(D) show the average relationship between the growth rates of COVID-19 cases (r_c, left panels) and deaths (r_d, right panels) and the city rank s for number of days since the first five daily cases (t_c) or deaths (t_d) and for different values of τ (indicated within the plots).

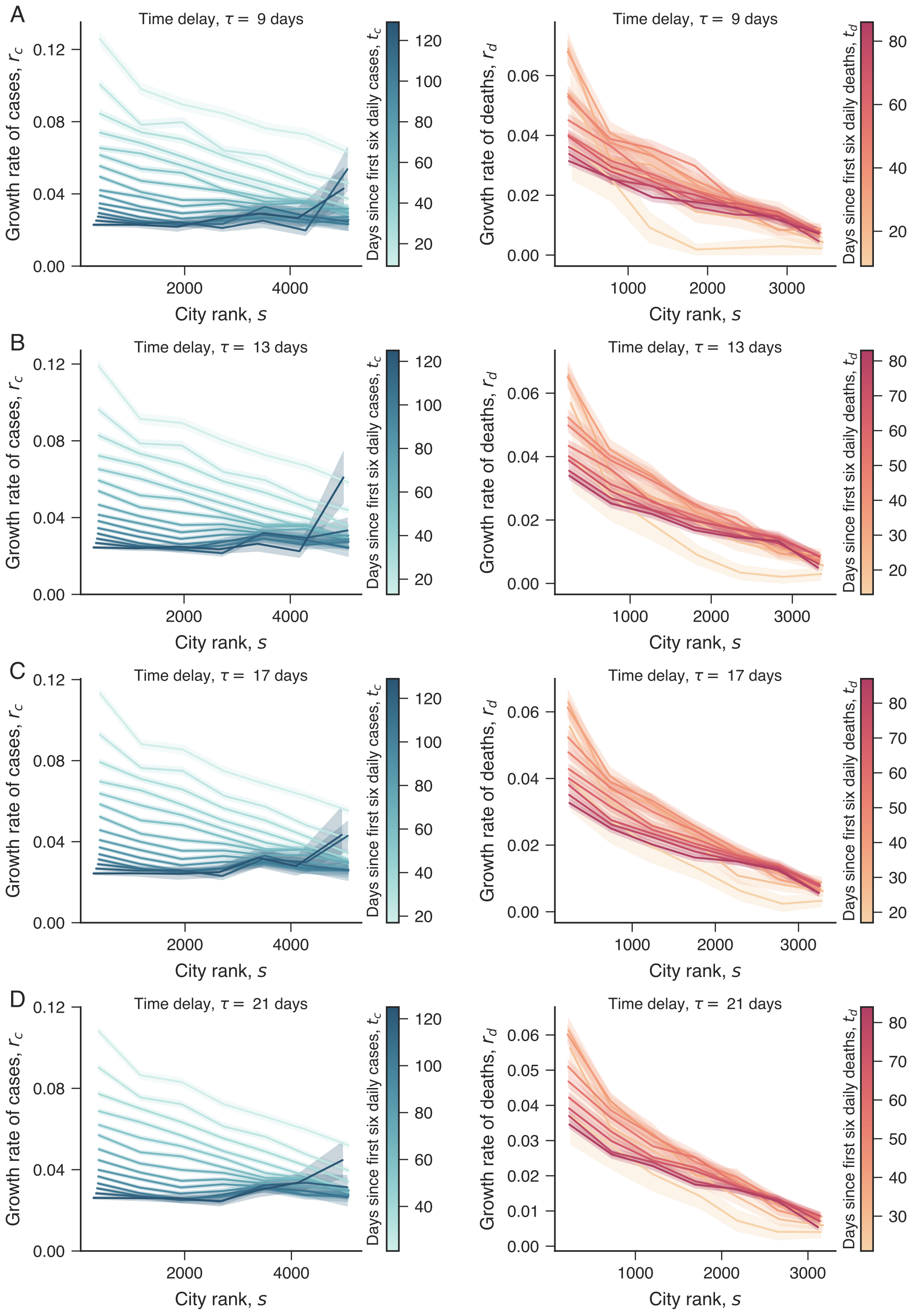

Figure 37. **Variations in the association between the growth rates and the city rank under different values of time delay τ.** Panels (A)-(D) show the average relationship between the growth rates of COVID-19 cases (r_c, left panels) and deaths (r_d, right panels) and the city rank s for number of days since the first six daily cases (t_c) or deaths (t_d) and for different values of τ (indicated within the plots).

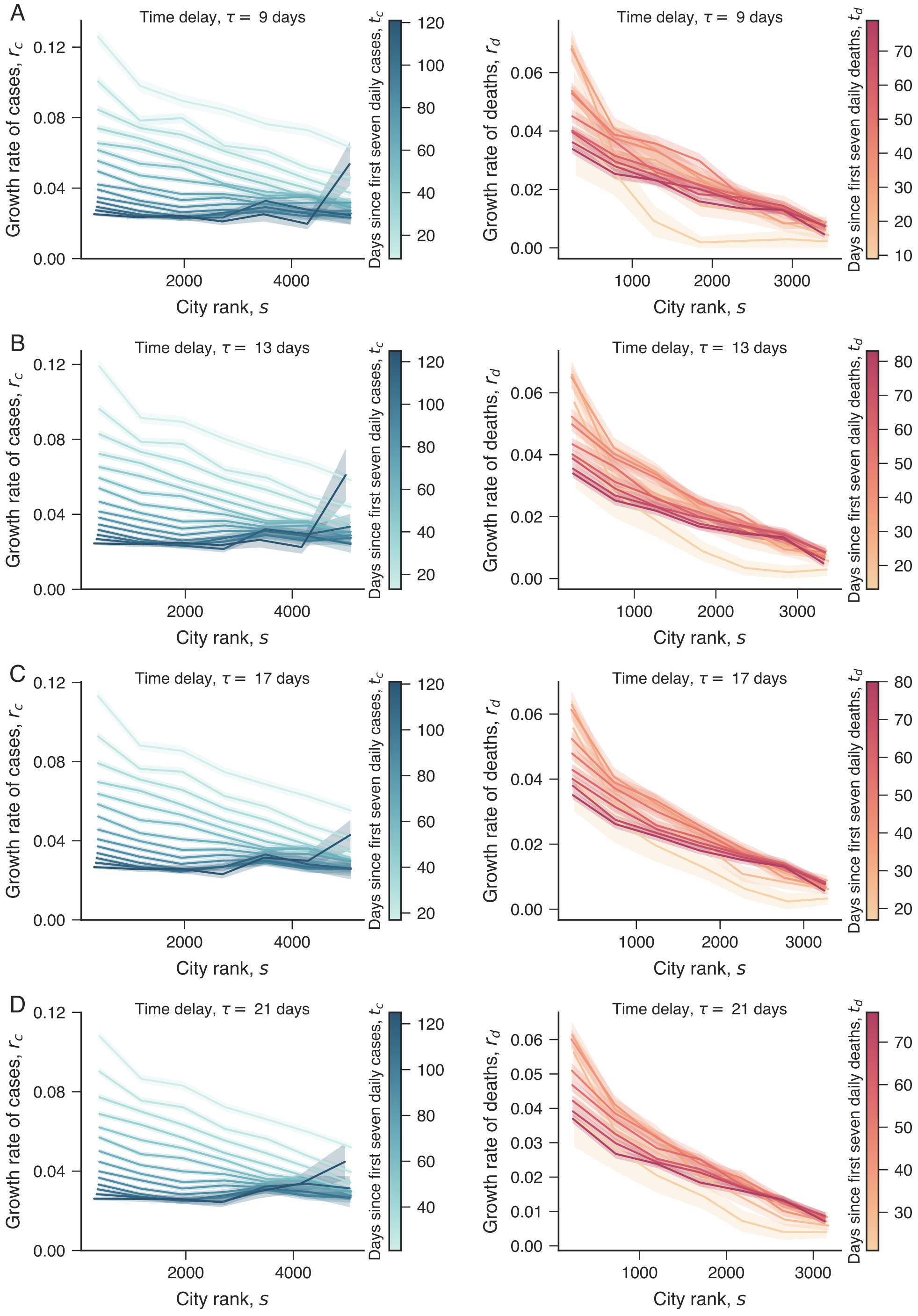

Figure 38. **Variations in the association between the growth rates and the city rank under different values of time delay τ.** Panels (A)-(D) show the average relationship between the growth rates of COVID-19 cases (r_c, left panels) and deaths (r_d, right panels) and the city rank s for number of days since the first seven daily cases (t_c) or deaths (t_d) and for different values of τ (indicated within the plots).
